# Patient and Prescriber Views of Penicillin Allergy Testing and Subsequent Antibiotic Use: A Rapid Review

**DOI:** 10.3390/antibiotics7030071

**Published:** 2018-08-06

**Authors:** Marta Wanat, Sibyl Anthierens, Christopher C. Butler, Judy M. Wright, Naila Dracup, Sue H. Pavitt, Jonathan A. T. Sandoe, Sarah Tonkin-Crine

**Affiliations:** 1 Nuffield Department of Primary Care Health Sciences, University of Oxford, Radcliffe Observatory Quarter, Woodstock Road, Oxford OX2 6GG, UK; christopher.butler@phc.ox.ac.uk (C.C.B.); sarah.tonkin-crine@ndm.ox.ac.uk (S.T.-C.); 2 Department of Primary and Interdisciplinary care, University of Antwerp, Campus “Drie Eiken”, Gebouw R, Universiteitsplein 1, B-2610 WILRIJK Antwerpen, Belgium; Sibyl.Anthierens@uantwerpen.be; 3 Leeds Institute of Health Sciences’, Faculty of Medicine and Health, University of Leeds, Worsley Building, Clarendon Way, Leeds LS2 9LU, UK; J.M.Wright@leeds.ac.uk (J.M.W.); N.Dracup@leeds.ac.uk (N.D.); 4 Dental Translational and Clinical Research Unit, Faculty of Medicine and Health, University of Leeds, Worsley Building, Clarendon Way, Leeds LS2 9LU, UK; S.Pavitt@leeds.ac.uk; 5 Healthcare Associated Infection Group, University of Leeds and Leeds Teaching Hospitals NHS Trust, Leeds LS13EX, UK; J.Sandoe@leeds.ac.uk; 6 NIHR Health Protection Research Unit in Healthcare Associated Infections and Antimicrobial Resistance, University of Oxford, Wellington Square, Oxford OX1 2JD, UK

**Keywords:** penicillin allergy, antibiotic stewardship, prescribing, antibiotic resistance

## Abstract

About 10% of U.K. patients believe that they are allergic to penicillin and have a “penicillin allergy label” in their primary care health record. However, around 90% of these patients may be mislabelled. Removing incorrect penicillin allergy labels can help to reduce unnecessary broad-spectrum antibiotic use. A rapid review was undertaken of papers exploring patient and/or clinician views and experiences of penicillin allergy testing (PAT) services and the influences on antibiotic prescribing behaviour in the context of penicillin allergy. We reviewed English-language publications published up to November 2017. Limited evidence on patients’ experiences of PAT highlighted advantages to testing as well as a number of concerns. Clinicians reported uncertainty about referral criteria for PAT. Following PAT and a negative result, a number of clinicians and patients remained reluctant to prescribe and consume penicillins. This appeared to reflect a lack of confidence in the test result and fear of subsequent reactions to penicillins. The findings suggest lack of awareness and knowledge of PAT services by both clinicians and patients. In order to ensure correct penicillin allergy diagnosis, clinicians and patients need to be supported to use PAT services and equipped with the skills to use penicillins appropriately following a negative allergy test result.

## 1. Introduction

Prescribing the most appropriate antibiotic class, the narrowest effective spectrum, duration, and dose will help to improve patient outcomes whilst reducing development of antibiotic resistance. Allergies to antibiotics influence prescribing decisions, sometimes preventing treatment with “first-line” antibiotics.

Penicillins are generally safe, effective, and narrow-spectrum and, as a result, are the first-line recommended treatment for many infections. However, about 10% of patients in the U.K. believe that they are allergic to penicillin and have a “penicillin allergy label” in primary care electronic health records [1]. Case series exploring the basis for these allergy labels reveal that only 10% of these people are likely to be truly allergic to penicillins [2]. As a result, these patients are usually prescribed alternative broad-spectrum antibiotics unnecessarily, which contributes to increased antimicrobial resistance. Non-penicillin antibiotics may be less effective treatments and carry additional long-term health risks [3]. Patients who are mislabeled with an allergy to penicillin can be readily identified using a combination of medical history, skin testing, and oral challenge [4]; this approach offers greater specificity than each element alone [5]. The combination of negative skin testing and negative oral challenge testing has been found to have >99% negative predictive value for penicillin allergy [6]. De-labelling has been found to be cost effective and safe in both children and adult populations [7,8].

The National Institute for Health and Care Excellence (NICE) has highlighted the importance of PAT and proposed criteria for selecting patients for testing [1]. Referral to, and use of, PAT services is not currently common practice in U.K. general practice and there is an unmet need for PAT services. Greater use of these services would help identify patients with incorrect allergy labels, provide access to a wider variety of antibiotic treatments for these patients, and potentially contribute to a reduction in prescribing of broad-spectrum antibiotics. In order to encourage use and expansion of such services the views and experiences of patients and clinicians need to be better understood to identify barriers and enablers to use.

A rapid review was undertaken to identify studies exploring patients’ and/or clinicians’ views and experiences of penicillin allergy testing (PAT) services and/or to explore the influences on antibiotic prescribing behaviour in the context of penicillin allergy. Rapid review is a form of synthesis to support review of existing evidence in a timely manner. It is particularly useful in healthcare where there is an increasing demand for a timely access to new evidence, especially for policymakers, healthcare professionals, and healthcare institutions [9,10]. Rapid reviews differ from systematic reviews in that they tend to have more targeted research questions; they often include limited sources with language and time restrictions, or use only one reviewer when selecting and extracting data [11].

## 2. Materials and Methods

We searched Applied Social Sciences Index and Abstracts (ASSIA) (ProQuest) 1987–present; the Cumulative Index to Nursing and Allied Health Literature (CINAHL) (EBSCO) 1981–present; Cochrane Central Register of Controlled Trials: Issue 10 of 12, October 2017 and Issue 11 of 12, November 2017; Cochrane Database of Systematic Reviews: Issue 10 of 12, October 2017 and Issue 11 of 12, November 2017; Database of Abstracts of Reviews of Effect: Issue 2 of 4, April 2015; Cochrane Methodology Register: Issue 3 of 4, July 2012; NHS Economic Evaluation Database: Issue 2 of 4, April 2015; Health Technology Assessment Database: Issue 4 of 4, October 2016; Embase Classic + Embase 1947 to 11 October 2017, Ovid MEDLINE(R) 1946 to November Week 1 2017, Ovid MEDLINE(R) Epub Ahead of Print 9 November 2017, Ovid MEDLINE(R) In-Process & Other Non-Indexed Citations 9 November 2017, PsycINFO 1806 to October Week 1 2017, PubMed (NLM) 1946—present, and Web of Science-Clarivate Analytics core collection. We searched for studies that described either (a) patient or clinician views and experiences of (diagnosing) penicillin allergy or testing for penicillin allergy or (b) influences on clinicians’ antibiotic prescribing behaviour in the context of penicillin allergy. Search strategies were developed for each database in collaboration with experienced information scientists (Supplementary Materials). In order to avoid missing potentially relevant studies, search strategies included terms related to “drug allergy” as well as “penicillin allergy”. Key search terms included penicillin allergy; penicillin testing; clinician/patient attitudes and experiences; drug prescribing; qualitative studies; drug allergy; and drug testing. Subject headings and free text words were identified for use in the search concepts by text analysis tools and project team members. Further terms were identified from relevant papers.

To be included in the review, papers had to focus on penicillin allergy (testing), explore the views of patients or clinicians (prescribers), and use qualitative or survey methods. One reviewer screened all abstracts against the inclusion criteria, with another screening 10% of the abstracts. This was followed by one reviewer screening the full texts of potentially eligible papers and extracting data from those that were eligible. Both reviewers agreed upon the final set of studies included. We examined the reference lists of all potentially relevant papers to identify any additional studies. We included all relevant papers which had an abstract written in English regardless of publication type.

## 3. Results

**Figure 1:**
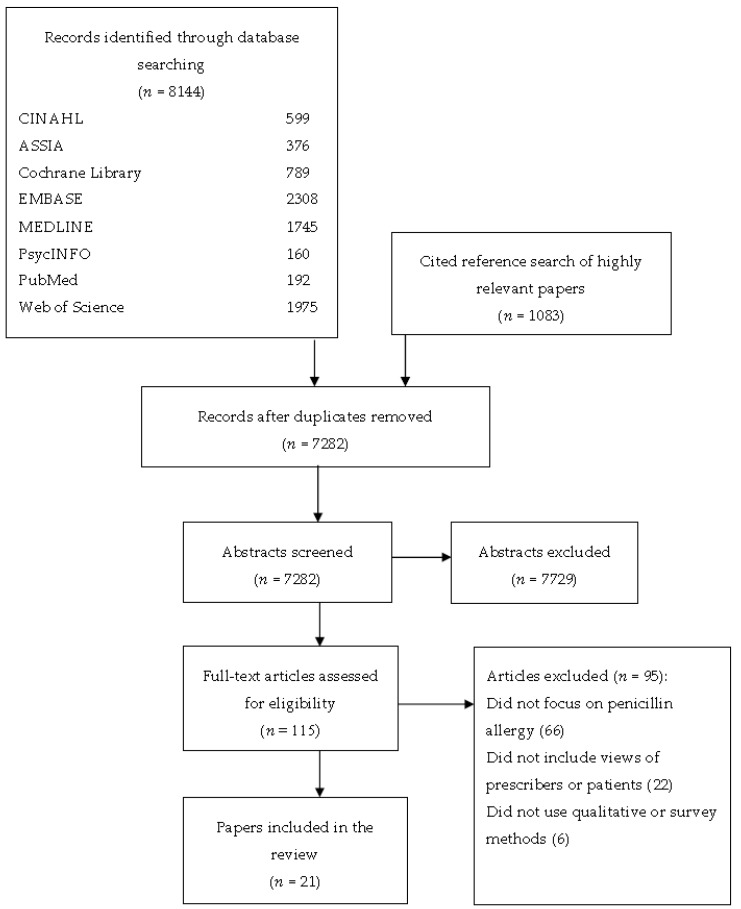
Flow diagram showing the identification of papers from database searches.

A total of 8144 papers were identified. Twenty-one articles were included in the final synthesis (Figure 1). Studies included participants from 10 countries, with 2 of the 21 studies set in the U.K. Ten studies investigated patients’ views, nine investigated clinicians’ views, while two focused on both patients’ and clinicians’ views. Two studies focused on the views of primary care clinicians, and nine on professionals working in secondary care. All of the studies used a cross-sectional survey design. An overview of the included studies is presented in Table 1.

### 3.1. Clinicians’ Views of Penicillin Allergy Testing

Six studies investigated clinicians’ referral rates for PAT as well as their views of the referral process. These included both primary and secondary care providers.

The referral rates reported by healthcare professionals were generally low. Picard et al. reported very low referral rates among clinicians in a Canadian hospital, where only 16% of clinicians had ever referred a patient to an allergist [24]. In a U.S. study, Sundquist and colleagues found that in a six-month period, primary care practitioners asked only 50% of eligible patients if they would like to attend PAT [30]. Two additional studies identified similar findings: Soni et al. found that more than 80% of general internal medicine practitioners in one U.S. health centre had never consulted an allergy service [27], whilst Elkhalifa et al. found that over 60% of health care professionals in a U.K. hospital had not referred to an allergy service in the past year [17]. Hayoun et al. asked French primary care practitioners who had referred patients for penicillin allergy testing what criteria they use for referring patients [21]. Over half reported no particular criteria for referring patients, and those who mentioned referral criteria reported various factors, thus suggesting a lack of a uniform approach. Reported criteria included initial severe reaction to beta-lactams, young age, and lack of alternative treatment options [21]. The criteria used to exclude patients from a diagnostic assessment were older age and reaction after eight days or maculopapular rash. Picard et al. also reported that one of the main barriers for clinicians to referring patients was not knowing indications for referral [24] while Soni et al. found that only 20% of clinicians were able to correctly identify appropriate patients for penicillin allergy skin testing [27].

Three studies focused specifically on barriers and facilitators to the referral. Sundquist et al. investigated views of primary care clinicians on referring patients to an allergy clinic. They were asked to indicate the degree to which possible barriers might prevent them from referring patients: the greatest perceived barrier was that patients may not want to get tested, followed by the time required for discussing testing in consultations; anticipation that patients would not want to risk having a reaction; forgetting to discuss testing; and physicians not being aware that patients had an allergy [30]. In a second study, Picard et al. reported that lack of allergy testing services in Canada was a major barrier to referral [24]. In addition, Trubiano et al. investigated views of adult and paediatric infectious disease physicians and found that 23% of respondents did not have access to any form of allergy testing whereas only 27% had access to skin testing combined with an oral challenge [31]. Also, 40% were unaware of the specific nature of available testing. In the same study, most respondents reported that it was worthwhile to refer patients for testing (93%), and they believed testing would lead to removing the antibiotic allergy label (78%). They also believed that potential benefits of removing antibiotic allergy label included better antibiotic selection, improved antibiotic appropriateness, and safety of antibiotic administration [31].

Lastly, Elkhalifa et al. highlighted difficulties for health care professionals in assessing allergy and reported that over 55% of clinicians thought that it was often impossible to draw conclusions based on history alone, especially given time constraints [17].

An additional five studies investigated practitioners’ views and knowledge of penicillin allergy, and found that health care professionals often had a limited knowledge of drug allergies. The studies reported that many healthcare professionals had misconceptions about the nature of allergy with four studies showing that 39–57% of hospital providers believed that allergy is permanent [13,15,20,27,28]. Amin et al. found that in a survey of hospital doctors, 63% of healthcare professionals were aware of true penicillin allergy reaction signs and symptoms, 23% had considered antibiotic side effects as an allergic reaction, and 13% were unaware of the characteristics of an allergic reaction [13]. Similarly, Blumenthal, who reported on knowledge levels of inpatient providers in a tertiary medical centre in the U.S., found that knowledge about penicillin skin testing was poor, with only 36% of providers knowing that skin testing is a valid tool for assessing penicillin allergy. In a study involving paediatric hospital providers, Grillo and Le reported that even fewer clinicians (21%) thought skin testing was a reliable tool for assessing pen-allergy [20]. Lastly, Amin et al. and Elkhalifa et al. highlighted that secondary care providers received little or no training in drug allergy and that they felt unprepared to deal with these allergies [13,17].

### 3.2. Patients’ Views of Penicillin Allergy Testing

Only two studies explored patients’ views and satisfaction with PAT. Sundquist and colleagues sought patients’ views following a three-step testing procedure consisting of skin prick testing, intradermal testing (IDT), and oral challenge (OC). These patients were referred by their primary care clinicians to a U.S. allergy clinic. They found that all patients felt that PAT provided valuable medical information [30]. However, this was measured using a single-item questionnaire (“Do you think undergoing testing for penicillin allergy provides important information for your penicillin history?”). Jose and Ishmael assessed patients’ views about hypothetical penicillin testing [22]. A small sample of patients attending a general allergy clinic in the U.S. were given a brief, 5-question survey to assess their knowledge and views of pen-allergy testing. The majority of respondents were unaware that drug allergy can wane over time, and they had not been informed by their primary care provider about the availability of PAT but all expressed an interest in testing [22].

### 3.3. Influences on Clinician Antibiotic Prescribing Behaviour when Treating Patients with Suspected Penicillin Allergy

Only two studies investigated influences on clinicians’ antibiotic prescribing behaviour in relation to penicillin allergy. Elkhalifa et al. found that almost 60% of consultants and doctors in training agreed that it is always better to “play it safe” and not use beta-lactams in patients labelled penicillin-allergic [13]. Interestingly, the majority did not agree with a statement that penicillin allergy is not important “as there are many alternative antibiotics to beta-lactams that have comparable efficacy and safety” [17]. However, Suetrong and Klaewsongkram surveyed Thai physicians and found that one of the major factors influencing prescribing behaviour was the ready availability of alternative antibiotics [29].

### 3.4. Influences on Clinician Antibiotic Prescribing Behaviour and Patient Antibiotic Use Following a Penicllin Allergy Test

Seven studies measured patient consumption of penicillins following a negative allergy test. Between 7% and 41% of patients continued to avoid penicillins following a negative test [12,16,18,19,23,25,26].

Studies investigated reasons for not taking medication after testing. However, investigators usually simply ascertained whether it was a patient who was reluctant to take penicillin or a clinician who was reluctant to prescribe the drug. While Eriksson et al. reported that it was mainly patients who were reluctant to take the drug [18], other studies reported that both patients were reluctant to take and general practitioners (GPs) were reluctant to prescribe penicillin [12,16,19,21,25]. Phillips and Gerace found that almost 50% of patients who reported continued avoidance of penicillin said that they would tell a new provider that they were allergic [19].

Eight studies investigated these reasons in more detail. One of the most commonly reported reasons among both patients and clinicians was fear of a further reaction, uncertainty about the severity of such a reaction, and lack of confidence that penicillin could be safely administered [12,14,18,23,32]. Eriksson et al. reported that patients were unsure which type of antibiotics they could use safely [18]. Gerace and Phillips found similar gaps in patients’ knowledge following a negative result. Although all patients were able to correctly identify whether skin testing had been positive or negative, patients who had had a negative result were often unsure which antibiotic class (penicillin and/or cephalosporins and/or all beta-lactams) they were now able to take [19]. Additional studies also reported that patients avoided penicillins either because of lack of confidence in the test results [14,16,23,26] or distrust of health care professionals’ advice [23]. Philips et al. reported that only 10% of patients felt that their primary care physician positively influenced their willingness to take beta-lactams after a negative allergy test [23]. Complementary to this, Suetrong and Klaewsongkram highlighted that only 5% of inpatient medical providers knew how to correctly interpret penicillin skin test results [29].

Finally, two studies investigated whether the type of allergy testing may affect patients’ confidence in the results. Erikson found that there was no difference in concerns between those who were investigated through skin test or through oral provocation test [18], and Ratzon found that while 100% of patients who tested negative by an extended drug provocation test (DPT) and later needed a beta-lactam used it, only 76% of patients who underwent short DPT actually took the drug when prescribed [25].

## 4. Discussion

This review provides an overview of the existing research in relation to patients’ and clinicians’ views of PAT and associated influences on antibiotic prescribing behaviour and penicillin use in the context of penicillin allergy.

There was limited evidence on patients’ views of PAT. Although it was found that patients valued the diagnostic information that tests provided, some thought it was a time-consuming process. Clinicians reported a number of barriers to referral which were related to occupational pressures, difficulties around taking allergy history, and their lack of knowledge about referral criteria as well as concerns about patient safety. Following a test, both patients and clinicians often remained reluctant to take and prescribe penicillins. This seemed to be linked to their negative beliefs about the test, including a lack of confidence in the test result, and fear of subsequent drug reactions.

The results suggest that discussing penicillin allergy and testing can be challenging for clinicians, especially if they are unsure about referral criteria and lack motivation to challenge a patient’s drug allergy status. None of the studies which focused on views of primary care clinicians mentioned antimicrobial stewardship or adherence to clinical guidelines for PAT as a reason for referring patients for PAT. This may be because the idea that PAT is a potentially important antimicrobial stewardship activity is relatively new and has not been well publicised in primary care. It is also possible that antimicrobial stewardship does not act as a sufficient motivator to change their behavior. The results also highlight that referring or attending for the test is not sufficient for both a patient and primary care clinician to accept the test result and prescribe/take penicillin following a negative test. Clinicians may require further information including the clinical meaning of a test result and its implications. Patient education could also include information about the test process and the potential benefits of being able to take penicillins. This could improve the patient’s confidence in the test results and their willingness to take the drug, should it be prescribed for them. However, this will only be possible if clinicians have adequate knowledge and understanding of allergies and testing. Both primary and secondary care professionals are likely to benefit from further training about penicillin allergy and testing, including its accuracy, implications, and benefits, in order to be able to discuss these issues with patients.

Changing both patients’ and GPs’ views about PAT and subsequent medication adherence is very important as it is in line with one of the key aims of antibiotic stewardship, as outlined in the U.K. five-year antimicrobial resistance (AMR) strategy to “conserve and steward the effectiveness of existing treatments” [33]. Penicillins are an important group of antibiotics and remain first-line therapy for many common infections but a record of penicillin allergy has significant effects for antimicrobial prescribing. Removing inappropriate penicillin allergy labels can help patients to receive first-line treatments for infections, suffer lesser side effects, and recover quicker [3,34,35]. It will also help in tacking antimicrobial resistance by substituting broad-spectrum antibiotics, which these patients normally receive, with narrow-spectrum penicillin.

### Limitations

The rapid review identified only studies which used a cross-sectional survey design to explore clinician and patient views, with the majority focusing on clinical aspects of penicillin allergy tests and their accuracy. No qualitative studies were identified. Therefore, exploring patients’ and clinicians’ motivations and beliefs was often only a secondary aim for most of the studies. As a result, the studies provided limited insight into patients’ and clinicians’ behaviour. This highlights the need for qualitative studies to understand these complex behaviours. As this was a rapid review, it is possible that with more time and resources a full systematic review could have found more studies using grey literature searches and including non-English-language reports. It is also possible that lack of quality assessment and lack of double-checking abstracts and full texts could have introduced a bias.

A limited number of studies explored clinicians’ reasons for not wanting to prescribe penicillins following a negative test. Most evidence came from studies with patients who reported their perspectives on their clinician’s difficulties in prescribing, rather than directly asking clinicians themselves. Subsequently, results should be interpreted with caution.

## 5. Conclusions

The existing literature highlights a number of challenges to successful implementation of PAT. Both patients and clinicians need to be supported to use PAT services and equipped with the skills to prescribe and consume penicillins appropriately following a negative test result.

## Figures and Tables

**Table 1:** Overview of the included studies.

Authors	Title	Country, Setting	Year	Design	Research Aim	Population
Al-Ahmad, M. & Rodriguez-Bouza, T. [12]	Drug allergy evaluation for beta-lactam hypersensitivity: Cross-reactivity with cephalosporins, carbapenems, and negative predictive value	Kuwait, tertiary public allergy centre	2017	Telephone survey	To evaluate subsequent beta-lactam use following a negative test	40 patients who had a negative penicillin allergy test
Amin, W., et al. [13] [Conference abstract]	A clinical audit on reporting and documentation of penicillin allergy at an NHS Foundation Trust Hospital	U.K., general hospital	2010	Questionnaire	To examine patients’ and professionals’ perceptions and knowledge regarding penicillin allergic reactions	30 hospital clinicians
Andres, B. et al. [14] [Conference abstract]	Suspected allergy to beta-lactam antibiotic: The value of diagnostic evaluation	Spain, Allergy Department	2013	Telephone survey	To assess the number of patients with confirmed beta-lactam allergy, drugs involved in reactions, and the usefulness of the diagnostic tests	40 patients who had a negative penicillin allergy test
Blumenthal, K.G. et al. [15]	Effect of a drug allergy educational program and antibiotic prescribing guideline on inpatient clinical providers’ antibiotic prescribing knowledge	U.S., tertiary care facility	2014	Questionnaire	To survey inpatient providers to ascertain their baseline drug allergy knowledge and preparedness in caring for patients with penicillin allergy	258 inpatient providers from neurology, paediatrics, internal medicine, orthopaedic surgery, general surgery, attending hospital physicians, and nurse practitioners
Cohen, S. et al. [16] [Conference abstract]	The real use of beta-lactams after “penicillin allergic” label removal	Israel, allergy clinic	2012	Telephone questionnaire	To assess patients’ confidence in their allergy test results and whether they have taken penicillin since testing	106 patients who had a negative penicillin allergy test
Elkhalifa, S. et al. [17] [Conference abstract]	Management of patients with a history of penicillin allergy: Barriers to best practice and strategies to overcome them	U.K., general hospital	2017	Email survey	To investigate prescribers’ knowledge of penicillin allergy diagnosis and management and their views about the barriers to correct management	164 hospital doctors including doctors in training, consultants, and non-medical prescribers
Eriksson, M. et al. [18] [Conference abstract]	Are patients prone to using penicillin after testing negative for penicillin allergy at a specialist centre?	Sweden, allergy clinic	2017	Questionnaire	To assess, if tested negatively for penicillin allergy, patients’ attitude to future penicillin treatment	103 patients who had a negative penicillin allergy test
Gerace, K. & Phillips, E. [19]	Penicillin allergy label persists despite negative testing	U.S., allergy clinic	2015	Email or telephone survey	To elucidate patient interpretation of their results and antibiotic utilisation after penicillin allergy testing (PAT)	42 patients who underwent PAT
Grillo, J. A. & Le, T.V. [20] [Conference abstract]	An assessment of current practice and knowledge of penicillin allergy at hospital-based paediatric centres	U.S., hospital-based centres	2015	Online survey	To assess current knowledge of clinicians and management of penicillin allergy	Inpatient paediatric providers at hospital-based centres
Hayoun, M.B. et al. [21]	The impact of allergy to beta-lactam antibiotics on general practitioners and patients in a cohort of 154 French patients	France, primary care	2015	Telephone questionnaire	To evaluate the role of the general practitioner (GP) in the management of allergy to Beta Lactams, and to analyse the interpretation of the allergological assessment by the GPs or patients themselves	80 GPs and 26 patients (when GP not available)
Jose, J. & Ishmael, F. T. [22] [Conference abstract]	A drug allergy education handout is an easy and effective method to improve patient awareness of penicillin allergy and increase penicillin testing	U.S., allergy clinic	2017	Survey	To assess patients’ prior knowledge of penicillin allergy and whether they were interested in being allergy tested	67 patients who attended a general allergy clinic
Phillips, E. J. et al. [23] [Conference abstract]	The Utility of Penicillin Skin Testing in a Tertiary Care Clinic	Canada, Drug Safety Clinic	2002	Telephone survey	To determine how the information provided from penicillin skin testing affects beta-lactam antibiotic use or patient attitudes towards future use of beta-lactam antibiotics	348 patients who had a negative penicillin allergy test
Picard, M. et al. [24]	Treatment of Patients with a History of Penicillin Allergy in a Large Tertiary-Care Academic Hospital	Canada, general hospital	2013	Survey	To assess allergy referral habits for patients with a history of penicillin allergy	44 attending physicians
Ratzon, R. et al., [25]	Impact of an extended challenge on the effectiveness of beta-lactam hypersensitivity investigation	Israel, medical centre	2016	Survey	To evaluate the effectiveness of a 7-day Drug Provocation Test (DPT) and a 1-day (short) DPT for beta-lactam allergy	49 patients who had a negative penicillin allergy test
Semedo, F.M. [26] [Conference abstract]	Full course drug provocation tests to penicillins—do we really need them?	Portugal, not known	2017	Survey	To evaluate diagnosis of penicillin allergy	54 patients who had a negative penicillin allergy test
Soni, D. et al. [27] [Conference abstract]	A clinical perspective: the prescribers’ true understanding of the penicillin-allergic patient	U.S., not known	2016	Survey	To survey knowledge of inpatient providers from different specialties regarding penicillin allergy	121 inpatients providers
Staicu, M.L. et al. [28]	A survey of inpatient practitioner knowledge of penicillin allergy at 2 community teaching hospitals	U.S., community-based teaching hospitals	2017	Online survey	To describe health care practitioner behaviour and identify potential knowledge gaps pertinent to the management of the penicillin-allergic patient	276 healthcare practitioners including attending physicians, advanced practice practitioners, pharmacists, and residents
Suetrong, N. & Klaewsongkram, J. [29]	The Differences and Similarities between Allergists and Non-Allergists for Penicillin Allergy Management	Thailand	2014	Email survey	To assess knowledge of penicillin allergy skin testing and attitudes towards the management of patients with a history of penicillin allergy	205 physicians including general practitioners, internists, paediatricians, allergists
Sundquist, B. K. et al. [30]	Proactive penicillin allergy testing in primary care patients labelled as allergic: outcomes and barriers	U.S., academic Internal Medicine practice	2017	Telephone survey (patients); Online survey (general practitioners)	To determine patient satisfaction with PAT to determine barriers to referring patients to testing	31 patients who had a negative penicillin allergy test 7 primary care clinicians who referred patients to the study
Trubiano, J. A. [31]	Improving Antimicrobial Stewardship by Antibiotic Allergy Delabelling: Evaluation of Knowledge, Attitude, and Practices Throughout the Emerging Infections Network	U.S. and Canada, the Emerging Infections Network	2016	Email survey	To assess receptiveness for incorporating antibiotic allergy testing in hospitals	736 active members of the Emerging Infections Network clinicians: Adult and paediatric Infectious Disease physicians
Warrington, R. J. et al. [32]	The value of skin testing for penicillin allergy in an inpatient population: analysis of the subsequent patient management.	Canada, allergy clinic	2000	Telephone survey	To assess why antibiotics were not taken after a negative skin test for penicillin allergy	84 patients who had a negative penicillin allergy skin test

## Supplementary Materials

The following are available online at http://www.mdpi.com/2079-6382/7/3/71/s1. Search Strategies.

## References

[1] National Institute for Health and Care Excellence (2014). Drug Allergy: Diagnosis and Management of Drug Allergy in Adults, Children and Young People.

[2] Minh H.-B.C., Bousquet P.J., Fontaine C., Kvedariene V., Demoly P. (2006). Systemic reactions during skin tests with β-lactams: A risk factor analysis. J. Allergy Clin. Immunol..

[3] Charneski L., Deshpande G., Smith S.W. (2011). Impact of an antimicrobial allergy label in the medical record on clinical outcomes in hospitalized patients. Pharmacotherapy.

[4] Mistry A., Arumugakani G., Toolan J., Ford K., Sandoe J., Wood P., Savic S. Does de-labelling penicillin allergy lead to a respective change in primary care records?. Proceedings of the British Society for Allergy and Clinical Immunology Annual Meeting.

[5] Salkind A.R., Cuddy P.G., Foxworth J.W. (2001). Is this patient allergic to penicillin? An evidence-based analysis of the likelihood of penicillin allergy. JAMA.

[6] Li J.T., Markus P.J., Osmon D.R., Estes L., Gosselin V.A., Hanssen A.D. (2000). Reduction of vancomycin use in orthopedic patients with a history of antibiotic allergy. Proc. Mayo Clin..

[7] Vyles D., Chiu A., Routes J., Castells M., Phillips E.J., Kibicho J., Brousseau D.C. (2018). Antibiotic Use After Removal of Penicillin Allergy Label. Pediatrics.

[8] Sacco K., Bates A., Brigham T., Imam J., Burton M. (2017). Clinical outcomes following inpatient penicillin allergy testing: A systematic review and meta-analysis. Allergy.

[9] Ganann R., Ciliska D., Thomas H. (2010). Expediting systematic reviews: Methods and implications of rapid reviews. Implement. Sci..

[10] Khangura S., Polisena J., Clifford T.J., Farrah K., Kamel C. (2014). Rapid review: An emerging approach to evidence synthesis in health technology assessment. Int. J. Technol. Assess. Health Care.

[11] Haby M.M., Chapman E., Clark R., Barreto J., Reveiz L., Lavis J.N. (2016). What are the best methodologies for rapid reviews of the research evidence for evidence-informed decision making in health policy and practice: A rapid review. Health Res. Policy Syst..

[12] Al-Ahmad M., Rodriguez-Bouza T. (2017). Drug allergy evaluation for betalactam hypersensitivity: Cross-reactivity with cephalosporines, carbapenems and negative predictive value. Asian Pac. J. Allergy Immunol..

[13] Amin W., Hitch G., Molai S., Khan I., Mulla R. (2010). A clinical audit on reporting and documentation of penicillin allergy at an NHS Foundation Trust Hospital. Int. J. Pharm. Pract..

[14] Andres B., Corominas M., Lleonart R. (2013). Suspected allergy to betalactam antibiotic: The value of diagnostic evaluation. Allergy.

[15] Blumenthal K.G., Shenoy E.S., Hurwitz S., Varughese C.A., Hooper D.C., Banerji A. (2014). Effect of a drug allergy educational program and antibiotic prescribing guideline on inpatient clinical providers’ antibiotic prescribing knowledge. J. Allergy Clin. Immunol. Pract..

[16] Cohen S., Khateeb-Alabbasi A., Nusem D., Panassof J. (2012). The real use of beta-lactams after “penicillin allergic” label removal. World Allergy Organ. J..

[17] Elkhalifa S., Calisti G., Owens L., Garcez T., Dodgson K., Alexander K. (2017). Management of patients with a history of penicillin allergy: Barriers to best practice and strategies to overcome them. Allergy.

[18] Eriksson M., Mincheva R., Pullerits T. (2017). Are patients prone to using penicillin after testing negative for penicillin allergy at a specialist centre?. Allergy.

[19] Gerace K., Phillips E. (2015). Penicillin allergy label persists despite negative testing. J. Allergy Clin. Immunol. Pract..

[20] Grillo J.A., Le T.V. (2015). An assessment of current practice and knowledge of penicillin allergy at hospital-based pediatric centers. J. Allergy Clin. Immunol..

[21] Hayoun M.B., Bourrier T., Pognonec C., Sanfiorenzo C., Marquette C., Leroy S. (2015). The impact of allergy to beta-lactam antibiotocs on general practitioners and patients in a cohort of154 French patients. Rev. Fr. Allergol..

[22] Jose J., Ishmael F.T. (2017). A drug allergy education handout is an easy and effective method to improve patient awareness of penicillin allergy and increase penicillin testing. J. Allergy Clin. Immunol..

[23] Phillips E.J., Knowles S.R., O’brien L., Weber E.A. (2002). The utility of penicillin skin testing in a tertiary care clinic. J. Allergy Clin. Immunol..

[24] Picard M., Bégin P., Bouchard H., Cloutier J., Lacombe-Barrios J., Paradis J., Des Roches A., Laufer B., Paradis L. (2013). Treatment of patients with a history of penicillin allergy in a large tertiary-care academic hospital. J. Allergy Clin. Immunol. Pract..

[25] Ratzon R., Reshef A., Efrati O., Deutch M., Forschmidt R., Cukierman-Yaffe T., Kenett R., Kidon M.I. (2016). Impact of an extended challenge on the effectiveness of beta-lactam hypersensitivity investigation. Ann. Allergy Asthma Immunol..

[26] Semedo F., Cruz C., Reis R., Tomaz E., Inacio F. (2017). Full course drug provocation tests to penicillins-do we really need them?. Allergy.

[27] Soni D., Ramsey A., Staicu M. (2016). A clinical perspective: The prescriber’s true understanding of the ‘penicillin allergic’patient. Ann. Allergy Asthma Immunol..

[28] Staicu M.L., Soni D., Conn K.M., Ramsey A. (2017). A survey of inpatient practitioner knowledge of penicillin allergy at 2 community teaching hospitals. Ann. Allergy Asthma Immunol..

[29] Suetrong N., Klaewsongkram J. (2014). The Differences and Similarities between Allergists and Non-Allergists for Penicillin Allergy Management. J. Allergy.

[30] Sundquist B.K., Bowen B.J., Otabor U., Celestin J., Sorum P.C. (2017). Proactive penicillin allergy testing in primary care patients labeled as allergic: Outcomes and barriers. Postgrad. Med..

[31] Trubiano J.A., Beekmann S.E., Worth L.J., Polgreen P.M., Thursky K.A., Slavin M.A., Grayson M.L., Phillips E.J. (2016). Improving Antimicrobial Stewardship by Antibiotic Allergy Delabeling: Evaluation of Knowledge, Attitude, and Practices Throughout the Emerging Infections Network. Open Forum Infect. Dis..

[32] Warrington R.J., Lee K.R., McPhillips S. (2000). The value of skin testing for penicillin allergy in an inpatient population: Analysis of the subsequent patient management. Allergy Asthma Proc..

[33] Department of Health and Social Care (2013). UK Five Year Antimicrobial Resistance Strategy 2013 to 2018.

[34] Arroliga M.E., Radojicic C., Gordon S.M., Popovich M.J., Bashour C.A., Melton A.L., Arroliga A.C. (2003). A prospective observational study of the effect of penicillin skin testing on antibiotic use in the intensive care unit. Infect. Control Hosp. Epidemiol..

[35] Nadarajah K., Green G.R., Naglak M. (2005). Clinical outcomes of penicillin skin testing. Ann. Allergy Asthma Immunol..

