# Supplementary material for: Patient and Prescriber Views of Penicillin Allergy Testing and Subsequent Antibiotic Use: A Rapid Review
Source: Antibiotics (Basel). 2018 Aug 6;7(3):71. doi: 10.3390/antibiotics7030071 (PMC6164736; doi:10.3390/antibiotics7030071)
Supplement: Supplementary file 1 [file antibiotics-07-00071-s001.pdf]

*Review*

# Patient and Prescriber Views of Penicillin Allergy Testing and Subsequent Antibiotic Use: A Rapid Review

**Marta Wanat [1,*], Sibyl Anthierens [2], Christopher C. Butler [1], Judy M. Wright [3], Naila Dracup [3], Sue H. Pavitt [4], Jonathan A.T. Sandoe [5] and Sarah Tonkin-Crine [1,6]**

1 Nuffield Department of Primary Care Health Sciences, University of Oxford, Radcliffe Observatory Quarter, Woodstock Road, Oxford OX2 6GG, UK; christopher.butler@phc.ox.ac.uk (C.C.B.); sarah.tonkin-crine@ndm.ox.ac.uk (S.T.-C.)
2 Department of Primary and Interdisciplinary care, University of Antwerp, Campus "Drie Eiken", Gebouw R, Universiteitsplein 1, B-2610 WILRIJK Antwerpen, Belgium; Sibyl.Anthierens@uantwerpen.be
3 Leeds Institute of Health Sciences', Faculty of Medicine and Health, University of Leeds, Worsley Building, Clarendon Way, Leeds LS2 9LU, UK; J.M.Wright@leeds.ac.uk (J.M.W.); N.Dracup@leeds.ac.uk (N.D.)
4 Dental Translational and Clinical Research Unit, Faculty of Medicine and Health, University of Leeds, Worsley Building, Clarendon Way, Leeds LS2 9LU, UK; S.Pavitt@leeds.ac.uk
5 Healthcare Associated Infection Group, University of Leeds and Leeds Teaching Hospitals NHS Trust, Leeds LS13EX, UK; J.Sandoe@leeds.ac.uk
6 NIHR Health Protection Research Unit in Healthcare Associated Infections and Antimicrobial Resistance, University of Oxford, Wellington Square, Oxford OX1 2JD, UK
* Correspondence: marta.wanat@phc.ox.ac.uk; Tel.: +44-1865-617-935

**Supplementary Materials**

**Search Strategies**

**Search 1: Patient or clinician views and experiences of diagnosing penicillin-allergy or testing for penicillin allergy**

**Applied Social Sciences Index and Abstracts (ASSIA) (ProQuest) 1987–present**

(SU.EXACT.EXPLODE ("Penicillin") AND SU.EXACT.EXPLODE("Side effects")) OR ti,ab((allerg* OR sensitiv* OR hypersensitiv* OR intoleran* OR anaphyla*) NEAR/5 penicillin*) OR ti,ab((adrs OR "adverse drug effect*" OR "adverse drug reaction*" OR "adverse effect*" OR "adverse event*" OR "adverse outcome*" OR "adverse reaction*") NEAR/5 penicillin*) 14 results

**CINAHL (EBSCO) 1981–present**

S37 S14 AND S36 95

S36 S15 OR S16 OR S17 OR S18 OR S19 OR S20 OR S21 OR S22 OR S23 OR S24 OR S25 OR S26 OR S27 OR S28 OR S29 OR S30 OR S31 OR S32 OR S33 OR S34 OR S35 1,609,075

S35 TX willing* 12,891

S34 (MH "Psychology, Social") 1,555

S33 (MH "Behavior+") 542,124

S32 (MH "Consumer Participation") 11,600

S31 TX (view* or experienc* or opinion* or attitude* or percep* or perceiv* or belie* or feel* or know* or understand*)  790,889

S30 TX (Qualitative or "grounded theory" or "phenomenological analysis" or "thematic analysis" or ethnograph* or "narrative analys*" or "phenomenological research")  119,294

S29 TX (questionnaire* or survey* or interview* or focus group* or case stud* or observ*)  892,070

S28 (MH "Interviews")  98,789

S27 (MH "Grounded Theory")  10,970

S26 (MH "Focus Groups")  26,254

S25 (MH "Questionnaires")  218,503

S24 (MH "Qualitative Studies")  64,651

S23 (MH "Attitude to Health+")  83,753

S22 (MH "Attitude of Health Personnel")  22,249

S21 (MH "Attitude")  8,759

S20 MW Ethic*  54,426

S19 (MH "Ethics+")  79,152

S18 (MH "Psychology")  3,638

S17 MW psychology  24,477

S16 (MH "Ethnic Groups+")  85,560

S15 (MH "Culture+")  108,250

S14 S7 AND S13  195

S13 S8 OR S9 OR S10 OR S11 OR S12  441,794

S12 (MH "Drug Hypersensitivity/DI")  463

S11 TI tolerability OR MW tolerability  1,373

S10 TI Test* OR MW Test*  440,467

S9 (MH "Skin Tests")  1,888

S8 TX (penicillin* N5 (test* or screen* or diagnos* or "re test*"))  155

S7 S1 OR S2 OR S3 OR S4 OR S5 OR S6  541

S6 TX ((adrs or adverse drug effect* or adverse drug reaction* or adverse effect* or adverse event* or adverse outcome* or adverse reaction*) N5 penicillin*)  241

S5 TX ((allerg* or sensitiv* or hypersensitiv* or intoleran* or anaphyla*) N5 penicillin*)  350

S4 (MH "Penicillins+") AND (MH "Anaphylaxis")  27

S3 (MH "Penicillins+") AND (MH "Adverse Drug Event")  28

S2 (MH "Penicillins/AE")  219

S1 (MM "Penicillins+") AND (MM "Drug Hypersensitivity+")  132

S25 S20 AND S24  109

S24 S21 OR S22 OR S23  373,064

S23 (MH "Questionnaires+")  224,694

S22 TI ( (interview* or questionnaire* or "focus group*") ) OR AB ( (interview* or questionnaire* or "focus group*") )  208,923

S21 TI ( (Qualitative or "grounded theory" or "phenomenological analysis" or "thematic analysis" or ethnograph* or "narrative analys*" or "phenomenological research") ) OR AB ( (Qualitative or "grounded theory" or "phenomenological analysis" or "thematic analysis" or ethnograph* or "narrative analys*" or "phenomenological research") )  73,586

S20 S10 AND S19  562

S19 S16 OR S17 OR S18  3,766

S18 TI ( ((Manage* or understand* or view* or experienc* or opinion* or attitude* or percep* or perceiv* or belie* or feel* or know* or understand* or fear* or expectation* or anxiety or anxious) N6 (drug hypersensitiv* or drug allerg*)) ) OR AB ( ((Manage* or understand* or view* or experienc* or opinion* or attitude* or percep* or perceiv* or belie* or feel* or know* or understand* or fear* or expectation* or anxiety or anxious) N6 (drug hypersensitiv* or drug allerg*)) )  62

S17 TI ( ((prescrib* or prescrip*) N8 ((Clinician* or physician* or doctor* or GP* or practitioner* or nurse* or "health professional*" or patient*) N6 (view* or experienc* or opinion* or attitude* or percep* or perceiv* or belie* or feel* or know* or understand* or fear* or expectation* or anxiety or anxious or influenc* or behavi* or barrier* or risk* or decision* or willing* or unwilling*))) ) OR AB ( ((prescrib* or prescrip*) N8 ((Clinician* or physician* or doctor* or GP* or practitioner* or nurse* or "health professional*" or patient*) N6 (view* or experienc* or opinion* or attitude* or percep* or perceiv* or belie* or feel* or know* or understand* or fear* or expectation* or anxiety or anxious or influenc* or behavi* or barrier* or risk* or decision* or willing* or unwilling*))) )  2,453

S16 S14 AND S15  1,411

S15 (MM "Practice Patterns" OR MM "Patient Satisfaction" OR MM "Attitude of Health Personnel+" OR MM "Attitude to Health" OR MM "Health Knowledge")  63,710

S14  S12 OR S13  109,995

S13  (MH "Prescriptions, Drug") OR (MH "Drugs, Prescription") OR (MH Prescribing patterns) OR (MH Inappropriate prescribing)  19,604

S12  S1 AND S11  92,588

S11  TX Administration and dosage*  158,238

S10  S5 OR S6 OR S7 OR S8 OR S9  73,627

S9  TI ( ((adrs or adverse) N3 (drug* or medication* or medicine* or antibiotic* or penicillin*)) ) OR AB ( ((adrs or adverse) N3 (drug* or medication* or medicine* or antibiotic* or penicillin*)) )  5,511

S8  TI ( ((allerg* or sensitiv* or hypersensitiv* or intoleran* or anaphyla*) N5 (drug* or medication* or medicine* or antibiotic* or penicillin*)) ) OR AB ( ((allerg* or sensitiv* or hypersensitiv* or intoleran* or anaphyla*) N5 (drug* or medication* or medicine* or antibiotic* or penicillin*)) ) 2,742

S7  (MH "Antiinfective Agents/AE" or MH "Antiinfective Agents/AE" or MH "Antiinflammatory Agents+/AE" or MH "Antineoplastic Agents+/AE" or MH "Antirheumatic Agents+/AE" or MH "Cardiovascular Agents+/AE" or MH "Central Nervous System Agents+/AE" or MH "Dermatologic Agents+/AE" or MH "Gastrointestinal Agents+ AE" or MH "Hematologic Agents+/AE" or MH " Antilipemic Agents+ AE" or MH "Radiation-Sensitizing Agents"+/AE" or MH "Renal Agents + AE" or MH "Reproductive Control Agent+/AE" OR MH "Drug Therapy/AE)  58,898

S6  (MM "Drug Hypersensitivity+")  2,169

S5  S1 AND S4  8,594

S4  S2 OR S3  11,072

S3  (MM "Adverse Drug Event+")  8,854

S2  (MH "Anaphylaxis")  2,250

S1  (MH "Antibiotics+" or MH "Antiinflammatory Agents+" or MH "Antineoplastic Agents" or MH "Antirheumatic Agents" or MH "Cardiovascular Agents+" or MH "Central Nervous System Agents+" or MH "Dermatologic Agents+" or MH "Gastrointestinal Agents+" or MH "Hematologic Agents+" or MH "Antilipemic Agents+" or MH "Radiation-Sensitizing Agents+" or MH "Renal Agents" or MH "Reproductive Control Agents+" OR MH "Drug Therapy+")  340,780

**Cochrane library:**

- **Cochrane Central Register of Controlled Trials : Issue 10 of 12, October 2017**
- **Cochrane Database of Systematic Reviews : Issue 11 of 12, November 2017**
- **Database of Abstracts of Reviews of Effect : Issue 2 of 4, April 2015**
- **Cochrane Methodology Register : Issue 3 of 4, July 2012**
- **Health Technology Assessment Database : Issue 4 of 4, October 2016**
- **Health Technology Assessment Database : Issue 4 of 4, October 2016**
- **About the Cochrane Collaboration : Issue 10 of 12, October 2017**

#1 MeSH descriptor: [Penicillins] explode all trees 4921

#2 MeSH descriptor: [Drug Hypersensitivity] explode all trees 935

#3 MeSH descriptor: [Drug-Related Side Effects and Adverse Reactions] this term only 1172

#4 MeSH descriptor: [Anaphylaxis] this term only 180

#5 MeSH descriptor: [Adverse Drug Reaction Reporting Systems] this term only 120

#6 {or #2-#5} 2347

#7 #1 and #6 75

#8 ((allerg* or sensitiv* or hypersensitiv* or intoleran* or anaphyla*) near/5 penicillin*):ti,ab,kw 278

#9 ((adrs or adverse drug effect* or adverse drug reaction* or adverse effect* or adverse event* or adverse outcome* or adverse reaction*) near/5 penicillin*):ti,ab,kw 122

#10 {or #7-#9} 440

#11 (penicillin* near/5 (test* or screen* or diagnos* or "re test*")):ti,ab,kw 202

#12 MeSH descriptor: [Skin Tests] this term only 1478

#13 test*:ti,kw 94969

#14 tolerability:ti,kw 18613

#15 {or #11-#13} 95012

#16 #10 and #15 162

#17 MeSH descriptor: [Culture] explode all trees 2695

#18 MeSH descriptor: [Ethnic Groups] explode all trees 3823

#19 MeSH descriptor: [Psychology] this term only 275

#20 MeSH descriptor: [Ethics] explode all trees 568

#21 MeSH descriptor: [Attitude] this term only 979

#22 MeSH descriptor: [Attitude to Health] this term only 2943

#23 MeSH descriptor: [Qualitative Research] this term only 795

#24 MeSH descriptor: [Surveys and Questionnaires] this term only 22207

#25 MeSH descriptor: [Focus Groups] this term only 460

#26 MeSH descriptor: [Grounded Theory] this term only 7

#27 (questionnaire* or survey* or interview* or focus group* or case stud* or observ*):ti,ab,kw 287721

#28 (Qualitative or "grounded theory" or "phenomenological analysis" or "thematic analysis" or ethnograph* or "narrative analys*" or "phenomenological research"):ti,ab,kw 8074

#29 (view* or experienc* or opinion* or attitude* or percep* or perceiv* or belie* or feel* or know* or understand*):ti,ab,kw 176415

#30 MeSH descriptor: [Patient Participation] this term only 1153

#31 MeSH descriptor: [Patient Acceptance of Health Care] this term only 2569

#32 MeSH descriptor: [Behavior] explode all trees 64189

#33 MeSH descriptor: [Psychology, Social] explode all trees 20269

#34 MeSH descriptor: [Interviews as Topic] this term only 1651

#35 willing*:ti,ab,kw 3881

#36 {or #17-#35} 429872

#37 #16 and #36 43

All Results (43)

Cochrane Reviews (0)
Other Reviews (0)
Trials (43)
Methods Studies (0)
Technology Assessments (0)
Economic Evaluations (0)
Cochrane Groups (0)

**Embase Classic+Embase 1947 to 2017 October 11**

1 exp *penicillin derivative/ (108266)

2 allergic reaction/ or exp allergy/ or exp drug hypersensitivity/ or anaphylaxis/ (184167)

3 1 and 2 (5318)

4 exp *penicillin derivative/ae [Adverse Drug Reaction] (6402)

5 exp *penicillin derivative/ and adverse drug reaction/ (6647)

6 exp *penicillin derivative/ and drug surveillance program/ (113)

7 ((allerg* or sensitiv* or hypersensitiv* or intoleran* or anaphyla*) adj5 penicillin*).tw. (7961)

8 ((adrs or adverse drug effect* or adverse drug reaction* or adverse effect* or adverse event* or adverse outcome* or adverse reaction*) adj5 penicillin*).tw. (195)

9 or/3-8 [Penicillin allergy] (21016)

10 (penicillin* adj5 (test* or screen* or diagnos* or "re test*")).tw. (3443)

11 skin test/ (41544)

12 (Test* adj skin).ti,kw. (58)

13 tolerability.ti,kw. (15497)

14 drug hypersensitivity/di [Diagnosis] (3471)

15 or/10-14 [Diagnosis or testing] (62169)

16 9 and 15 [pen allergy and sensitivity testing] (2330)

17 exp ethnic group/ (120866)

18 exp ethics/ (258066)

19 Ethic*.tw. (145226)

20 attitude/ (59249)

21 health personnel attitude/ (73408)

22 exp attitude to health/ (97727)

23 qualitative research/ (48845)

24 questionnaire/ (554326)

25 grounded theory/ (4490)

26 interview/ (169716)

27 (questionnaire* or survey* or interview* or focus group* or case stud* or observ*).tw. (5357381)

28 (Qualitative or "grounded theory" or "phenomenological analysis" or "thematic analysis" or ethnograph* or "narrative analys*" or "phenomenological research").tw. (238325)

29 (view* or experienc* or opinion* or attitude* or percep* or perceiv* or belie* or feel* or know* or understand*).tw. (5354685)

30 patient participation/ (22689)

31 patient attitude/ (59237)

32 exp *behavior/ (1359621)

33 social psychology/ (77715)

34 willing*.tw. (45780)

35 or/17-34 [Attitudes and indicators of attitude studies] (10469690)

36 16 and 35 [Attiudes and Penicillin allergy testing] (597)

**Ovid MEDLINE(R) 1946 to November Week 1 2017**

1 exp *Penicillins/ and exp *Drug Hypersensitivity/ (2070)

2 exp *Penicillins/ae (4000)

3 exp Penicillins/ and "drug-related side effects and adverse reactions"/ (201)

4 exp Penicillins/ and Anaphylaxis/ (708)

5 exp Penicillins/ and Adverse Drug Reaction Reporting Systems/ (37)

6 ((allerg* or sensitiv* or hypersensitiv* or intoleran* or anaphyla*) adj5 penicillin*).tw. (4534)

7 ((adrs or adverse drug effect* or adverse drug reaction* or adverse effect* or adverse event* or adverse outcome* or adverse reaction*) adj5 penicillin*).tw. (135)

8 or/1-7 [Penicillin allergy] (8125)

9 (penicillin* adj5 (test* or screen* or diagnos* or "re test*")).tw. (2005)

10 Skin Tests/ (34488)

11 test*.ti,kw. (374682)

12 tolerability.ti,kw. (7976)

13 exp Drug Hypersensitivity/di (8579)

14 or/9-13 [Diagnosis or testing] (417668)

15 8 and 14 [pen allergy or sensitivity testing] (1482)

16 exp Culture/ (155260)

17 exp Ethnic Groups/ (147027)

18 px.fs. (980336)

19 es.fs. (66985)

20 exp Ethics/ (144944)

21 attitude/ or "attitude of health personnel"/ or exp attitude to health/ (521032)

22 Qualitative Research/ (38842)

23 "Questionnaires"/ (418435)

24 Focus Group/ (25318)

25 Grounded Theory/ (762)

26 Interviews as topic/ (56925)

27 (questionnaire* or survey* or interview* or focus group* or case stud* or observ*).tw. (3798891)

28 (Qualitative or "grounded theory" or "phenomenological analysis" or "thematic analysis" or ethnograph* or "narrative analys*" or "phenomenological research").tw. (171661)

29 (view* or experienc* or opinion* or attitude* or percep* or perceiv* or belie* or feel* or know* or understand*).tw. (3784376)

30 Patient Participation/ (23549)

31 "Patient Acceptance of Health Care"/ (41338)

32 exp Behavior/ (1588047)

33 exp Psychology, Social/ (839685)

34 willing*.tw. (30191)

35 or/16-34 [Attitudes and indicators of attitude studies] (8397120)

36 15 and 35 [Attiudes and Penicillin allergy testing] (296)

**Ovid MEDLINE(R) Epub Ahead of Print November 09, 2017**

1 exp *Penicillins/ and exp *Drug Hypersensitivity/ (0)

2 exp *Penicillins/ae (0)

3 exp Penicillins/ and "drug-related side effects and adverse reactions"/ (0)

4 exp Penicillins/ and Anaphylaxis/ (0)

5 exp Penicillins/ and Adverse Drug Reaction Reporting Systems/ (0)

6 ((allerg* or sensitiv* or hypersensitiv* or intoleran* or anaphyla*) adj5 penicillin*).tw. (36)

7 ((adrs or adverse drug effect* or adverse drug reaction* or adverse effect* or adverse event* or adverse outcome* or adverse reaction*) adj5 penicillin*).tw. (1)

8 or/1-7 [Penicillin allergy] (37)

9 (penicillin* adj5 (test* or screen* or diagnos* or "re test*")).tw. (13)

10 Skin Tests/ (0)

11 test*.ti,kw. (6045)

12 tolerability.ti,kw. (165)

13 exp Drug Hypersensitivity/di (0)

14 or/9-13 [Diagnosis or testing] (6217)

15 8 and 14 [pen allergy or sensitivity testing] (6)

16 exp Culture/ (1)

17 exp Ethnic Groups/ (0)

18 px.fs. (1)

19 es.fs. (0)

20 exp Ethics/ (0)

21 attitude/ or "attitude of health personnel"/ or exp attitude to health/ (0)

22 Qualitative Research/ (0)

23 "Questionnaires"/ (1)

24 Focus Group/ (0)

25 Grounded Theory/ (0)

26 Interviews as topic/ (0)

27 (questionnaire* or survey* or interview* or focus group* or case stud* or observ*).tw. (84164)

28 (Qualitative or "grounded theory" or "phenomenological analysis" or "thematic analysis" or ethnograph* or "narrative analys*" or "phenomenological research").tw. (7130)

29 (view* or experienc* or opinion* or attitude* or percep* or perceiv* or belie* or feel* or know* or understand*).tw. (93274)

30 Patient Participation/ (0)

31 "Patient Acceptance of Health Care"/ (0)

32 exp Behavior/ (0)

33 exp Psychology, Social/ (1)

34 willing*.tw. (1033)

35 or/16-34 [Attitudes and indicators of attitude studies] (151674)

36 15 and 35 [Attiudes and Penicillin allergy testing] (2)

**Ovid MEDLINE(R) In-Process & Other Non-Indexed Citations November 09, 2017**

1 exp *Penicillins/ and exp *Drug Hypersensitivity/ (0)

2 exp *Penicillins/ae (0)

3 exp Penicillins/ and "drug-related side effects and adverse reactions"/ (0)

4 exp Penicillins/ and Anaphylaxis/ (0)

5 exp Penicillins/ and Adverse Drug Reaction Reporting Systems/ (0)

6 ((allerg* or sensitiv* or hypersensitiv* or intoleran* or anaphyla*) adj5 penicillin*).tw. (205)

7 ((adrs or adverse drug effect* or adverse drug reaction* or adverse effect* or adverse event* or adverse outcome* or adverse reaction*) adj5 penicillin*).tw. (8)

8 or/1-7 [Penicillin allergy] (210)

9 (penicillin* adj5 (test* or screen* or diagnos* or "re test*")).tw. (118)

10 Skin Tests/ (0)

11 test*.ti,kw. (28797)

12 tolerability.ti,kw. (1060)

13 exp Drug Hypersensitivity/di (0)

14 or/9-13 [Diagnosis or testing] (29935)

15 8 and 14 [pen allergy or sensitivity testing] (40)

16 exp Culture/ (5)

17 exp Ethnic Groups/ (7)

18 px.fs. (56)

19 es.fs. (3)

20 exp Ethics/ (8)

21 attitude/ or "attitude of health personnel"/ or exp attitude to health/ (5)

22 Qualitative Research/ (0)

23 "Questionnaires"/ (8)

24 Focus Group/ (0)

25 Grounded Theory/ (0)

26 Interviews as topic/ (0)

27 (questionnaire* or survey* or interview* or focus group* or case stud* or observ*).tw. (453413)

28 (Qualitative or "grounded theory" or "phenomenological analysis" or "thematic analysis" or ethnograph* or "narrative analys*" or "phenomenological research").tw. (27550)

29 (view* or experienc* or opinion* or attitude* or percep* or perceiv* or belie* or feel* or know* or understand*).tw. (472683)

30 Patient Participation/ (1)

31 "Patient Acceptance of Health Care"/ (0)

32 exp Behavior/ (92)

33 exp Psychology, Social/ (72)

34 willing*.tw. (3942)

35 or/16-34 [Attitudes and indicators of attitude studies] (809216)

36 15 and 35 [Attiudes and Penicillin allergy testing] (9)

**PsycINFO 1806 to October Week 1 2017**

1 exp PENICILLINS/ (146)

2 exp "Side Effects (Drug)"/ or exp "Side Effects (Treatment)"/ or exp Anaphylactic Shock/ or exp DRUG ALLERGIES/ (53979)

3 1 and 2 (13)

4 ((allerg* or sensitiv* or hypersensitiv* or intoleran* or anaphyla*) adj5 penicillin*).tw. (15)

5 ((adrs or adverse drug effect* or adverse drug reaction* or adverse effect* or adverse event* or adverse outcome* or adverse reaction*) adj5 penicillin*).tw. (2)

6 or/3-5 [Penicillin allergy] (27)

7 (test* or screen* or diagnos* or "re test*").tw. (1005666)

8 Test*.ti,id. (176107)

9 tolerability.ti,id. (2417)

10 exp DIAGNOSIS/ (159844)

11 exp SCREENING/ (21906)

12 or/7-11 (1073764)

13 6 and 12 (11)

**PubMed (NLM) 1946 - present**

((("Drug Hypersensitivity"[Mesh]) AND "Penicillins"[Mesh])) AND ((pubstatusaheadofprint OR publisher[sb] OR pubmednotmedline[sb])) 0 results

(((adrs or adverse drug effect* or adverse drug reaction* or adverse effect* or adverse event* or adverse outcome* or adverse reaction*[tiab])) AND Penicillin*[Title/Abstract]) AND ((pubstatusaheadofprint OR publisher[sb] OR pubmednotmedline[sb])) 74 results

**Web of Science - Clarivate Analytics:**

- **Arts & Humanities Citation Index (Clarivate Analytics Web of Science) 1975-present**
- **Book Citation Index– Social Sciences & Humanities (Clarivate Analytics Web of Science) 2005-present**
- **Conference Proceedings Citation Index- Science (Clarivate Analytics Web of Science) 1990-present**
- **Conference Proceedings Citation Index- Social Science & Humanities (Clarivate Analytics Web of Science) 1990-present**
- **Sciences Citation Index (Clarivate Analytics Web of Science) 1900-present**
- **Social Sciences Citation Index (Clarivate Analytics Web of Science) 1900-present**
- **Web of Science Core Collection: Citation Indexes (Clarivate Analytics) 1900-present**
- **Emerging Sources Citation Index (ESCI) --2015-present**

# 22 #21 AND #8 337

Indexes=SCI-EXPANDED, SSCI, A&HCI, CPCI-S, CPCI-SSH, ESCI Timespan=1900-2017

# 21 #20 OR #19 OR #18 OR #17 OR #16 OR #15 OR #14 OR #13 OR #12 OR #11 OR #10 OR #9 15,780,209

Indexes=SCI-EXPANDED, SSCI, A&HCI, CPCI-S, CPCI-SSH, ESCI Timespan=1900-2017

# 20 ts=(Willing*) 62,430

Indexes=SCI-EXPANDED, SSCI, A&HCI, CPCI-S, CPCI-SSH, ESCI Timespan=1900-2017

# 19 ts=(Social psycholog*) 94,810

# 18 ts=(Behavio$r) 2,878,451

Indexes=SCI-EXPANDED, SSCI, A&HCI, CPCI-S, CPCI-SSH, ESCI Timespan=1900-2017

# 17 TS=(Patient near/2 (acceptance or participat*)) 22,182

Indexes=SCI-EXPANDED, SSCI, A&HCI, CPCI-S, CPCI-SSH, ESCI Timespan=1900-2017

# 16 ts=(view* or experienc* or opinion* or attitude* or percep* or perceiv* or belie* or feel* or know* or understand*) 7,003,469

Indexes=SCI-EXPANDED, SSCI, A&HCI, CPCI-S, CPCI-SSH, ESCI Timespan=1900-2017

# 15 ts=(Qualitative or "grounded theory" or "phenomenological analysis" or "thematic analysis" or ethnograph* or "narrative analys*" or "phenomenological research") 394,931

Indexes=SCI-EXPANDED, SSCI, A&HCI, CPCI-S, CPCI-SSH, ESCI Timespan=1900-201

# 14 ts=(questionnaire* or survey* or interview* or focus group* or case stud* or observ*) 7,724,795

Indexes=SCI-EXPANDED, SSCI, A&HCI, CPCI-S, CPCI-SSH, ESCI Timespan=1900-2017

# 13 TS=Attitud* 295,748

Indexes=SCI-EXPANDED, SSCI, A&HCI, CPCI-S, CPCI-SSH, ESCI Timespan=1900-2017

# 12 ts=Ethic* 178,996

Indexes=SCI-EXPANDED, SSCI, A&HCI, CPCI-S, CPCI-SSH, ESCI Timespan=1900-2017

# 11 TS=Psycholog* 410,604

Indexes=SCI-EXPANDED, SSCI, A&HCI, CPCI-S, CPCI-SSH, ESCI Timespan=1900-2017

# 10 ts=(Ethnic Group*) 54,981

Indexes=SCI-EXPANDED, SSCI, A&HCI, CPCI-S, CPCI-SSH, ESCI Timespan=1900-2017

# 9 TS=(Culture or cultural) 1,510,950

Indexes=SCI-EXPANDED, SSCI, A&HCI, CPCI-S, CPCI-SSH, ESCI Timespan=1900-2017

# 8 #3 AND #7 814

Indexes=SCI-EXPANDED, SSCI, A&HCI, CPCI-S, CPCI-SSH, ESCI Timespan=1900-2017

# 7 #4 OR #5 OR #6 135,489

Indexes=SCI-EXPANDED, SSCI, A&HCI, CPCI-S, CPCI-SSH, ESCI Timespan=1900-2017

# 6 ts=Tolerability 47,653

Indexes=SCI-EXPANDED, SSCI, A&HCI, CPCI-S, CPCI-SSH, ESCI Timespan=1900-2017

# 5 TS=Skin test* 86,990

Indexes=SCI-EXPANDED, SSCI, A&HCI, CPCI-S, CPCI-SSH, ESCI Timespan=1900-2017

# 4 TS=(penicillin* near/5 (test* or screen* or diagnos* or "re test*")) 1,747

Indexes=SCI-EXPANDED, SSCI, A&HCI, CPCI-S, CPCI-SSH, ESCI Timespan=1900-2017

# 3 #1 OR #2 3,681

Indexes=SCI-EXPANDED, SSCI, A&HCI, CPCI-S, CPCI-SSH, ESCI Timespan=1900-2017

# 2 TS=((adrs or "adverse drug effect*" or "adverse drug reaction*" or "adverse effect*" or "adverse event*" or "adverse outcome*" or "adverse reaction*") near/5 penicillin*) 112

Indexes=SCI-EXPANDED, SSCI, A&HCI, CPCI-S, CPCI-SSH, ESCI Timespan=1900-2017

# 1 ts=((allerg* or sensitiv* or hypersensitiv* or intoleran* or anaphyla*) near/5 penicillin*)

Indexes=SCI-EXPANDED, SSCI, A&HCI, CPCI-S, CPCI-SSH, ESCI Timespan=1900-2017
3,606

**Search 2: Patient or clinician views and experiences of diagnosing drug-allergy or testing for drug allergy**

**Applied Social Sciences Index and Abstracts (ASSIA) (ProQuest) 1987- present**

(ti,ab((Drug OR medication OR medicine) NEAR/3 (test OR tolerability OR screen OR diagnos* OR "re test*")) OR (MAINSUBJECT.EXACT("Hypersensitivity") AND MAINSUBJECT.EXACT.EXPLODE("diagnosis"))) AND ((MAINSUBJECT.EXACT("Drugs") OR MAINSUBJECT.EXACT.EXPLODE("Penicillin") OR MAINSUBJECT.EXACT.EXPLODE("Antibiotics")) AND (MAINSUBJECT.EXACT("Anaphylaxis") OR MAINSUBJECT.EXACT.EXPLODE("Side effects") OR MAINSUBJECT.EXACT.EXPLODE("Critical incidents")))

**CINAHL (EBSCO) 1981- present**

S33 S14 AND S32 395

SS32 S15 OR S16 OR S17 OR S18 OR S19 OR S20 OR S21 OR S22 OR S23 OR S24 OR S25 OR S26 OR S27 OR S28 OR S29 OR S30 OR S31 1,210,063

SS31 TI willing* OR AB willing* 9,183

SS30 (MH "Psychology, Social+") 708,657

SS29 (MH "Consumer Participation") 11,637

SS28 TI (((Clinician* or physician* or doctor* or GP* or practitioner* or nurse* or "health professional*" or patient*) n5 (view* or experienc* or opinion* or attitude* or percep* or perceiv* or belie* or feel* or know* or understand*)))OR AB (((Clinician* or physician* or doctor* or GP* or practitioner* or nurse* or "health professional*" or patient*) n5 (view* or experienc* or opinion* or attitude* or percep* or perceiv* or belie* or feel* or know* or understand*)) ) 118,899

SS27 TI ((Qualitative or "grounded theory" or "phenomenological analysis" or "thematic analysis" or ethnograph* or "narrative analys*" or "phenomenological research"))OR AB ((Qualitative or

"grounded theory" or "phenomenological analysis" or "thematic analysis" or ethnograph* or "narrative analys*" or "phenomenological research") ) 74,174

SS26 TI ((questionnaire* or survey* or interview* or focus group* or case stud* or observ*))OR AB ((questionnaire* or survey* or interview* or focus group* or case stud* or observ*) ) 487,501

SS25 (MH "Interviews") 99,051

SS24 (MH "Grounded Theory") 11,018

SS23 (MH "Focus Groups") 26,377

SS22 (MH "Questionnaires") 219,272

SS21 (MH "Qualitative Studies") 64,960

SS20 (MH "Attitude to Health") 19,971

SS19 (MH "Attitude of Health Personnel") 22,352

SS18 (MH "Attitude") 8,801

SS17 (MH "Ethics+") 79,320

SS16 (MH "Ethnic Groups+") 85,823

SS15 (MH "Culture+") 108,592

SS14 S10 AND S13 1,190

SS13 S11 OR S12 7,174

SS12 (MH "Drug Hypersensitivity/DI") 464

SS11 TI (((Drug* or medication* or medicine*) N3 (test* or tolerability or screen* or diagnos* or "re test*")))OR AB (((Drug* or medication* or medicine*) N3 (test* or tolerability or screen* or diagnos* or "re test*")) ) 6,760

SS10 S5 OR S6 OR S7 OR S8 OR S9 73,924

SS9 TI (((adrs or adverse) N3 (drug* or medication* or medicine* or antibiotic* or penicillin*)))OR AB (((adrs or adverse) N3 (drug* or medication* or medicine* or antibiotic* or penicillin*)) ) 5,541

SS8 TI (((allerg* or sensitiv* or hypersensitiv* or intoleran* or anaphyla*) N5 (drug* or medication* or medicine* or antibiotic* or penicillin*)))OR AB (((allerg* or sensitiv* or hypersensitiv* or intoleran* or anaphyla*) N5 (drug* or medication* or medicine* or antibiotic* or penicillin*)) ) 2,758

SS7 (MH "Antiinfective Agents/AE" or MH "Antiinflammatory Agents+/AE" or MH "Antineoplastic Agents+/AE" or MH "Antirheumatic Agents+/AE" or MH "Cardiovascular Agents+/AE" or MH "Central Nervous System Agents+/AE" or MH "Dermatologic Agents+/AE" or MH "Gastrointestinal Agents+ AE" or MH "Hematologic Agents+/AE" or MH " Antilipemic Agents+ AE" or MH "Radiation-

Sensitizing Agents"+/AE" or MH "Renal Agents + AE" or MH "Reproductive Control Agent+/AE" OR MH "Drug Therapy/AE) 59,067

SS6 (MM "Drug Hypersensitivity+") 2,176

SS5 S1 AND S4 8,702

SS4 S2 OR S3 11,112

SS3 (MM "Adverse Drug Event+") 8,892

SS2 (MH "Anaphylaxis") 2,253

SS1 (MH "Antiinfective Agents+"or MH "Antiinflammatory Agents+" or MH "Antineoplastic Agents" or MH "Antirheumatic Agents" or MH "Cardiovascular Agents+" or MH "Central Nervous System Agents+" or MH "Dermatologic Agents+" or MH "Gastrointestinal Agents+" or MH "Hematologic Agents+" or MH "Antilipemic Agents+" or MH "Radiation-Sensitizing Agents+" or MH "Renal Agents" or MH "Reproductive Control Agents+" OR MH "Drug Therapy+") 372,014

**Cochrane library**

Cochrane Central Register of Controlled Trials : Issue 10 of 12, October 2017
Cochrane Database of Systematic Reviews : Issue 11 of 12, November 2017
Database of Abstracts of Reviews of Effect : Issue 2 of 4, April 2015

ID Search Hits

#1 MeSH descriptor: [Therapeutic Uses] explode all trees 155036

#2 MeSH descriptor: [Anaphylaxis] this term only 181

#3 MeSH descriptor: [Drug-Related Side Effects and Adverse Reactions] explode all trees 3053

#4 #2 or #3 3209

#5 #1 and #4 1477

#6 MeSH descriptor: [Drug Hypersensitivity] this term only 446

#7 ((allerg* or sensitiv* or hypersensitiv* or intoleran* or anaphyla*) near/5 (drug* or medication* or medicine* or antibiotic* or penicillin*)):ti,ab 3300

#8 ((adrs or adverse) near/3 (drug* or medication* or medicine* or antibiotic* or penicillin*)):ti,ab 5160

#9 #5 or #6 or #7 or #8 9855

#10 ((Drug* or medication* or medicine*) near/3 (test* or tolerability or screen* or diagnos* or "re test*")):ti,ab 7348

#11 MeSH descriptor: [Drug Hypersensitivity] explode all trees 943

#12 MeSH descriptor: [Diagnosis] explode all trees 316120

#13 #11 and #12 476

#14 #10 or #13 7795

#15 #9 and #14 917

#16 MeSH descriptor: [Culture] explode all trees 2727

#17 MeSH descriptor: [Ethnic Groups] explode all trees 3871

#18 MeSH descriptor: [Ethics] explode all trees 579

#19 MeSH descriptor: [Attitude] this term only 991

#20 MeSH descriptor: [Attitude of Health Personnel] this term only 1841

#21 MeSH descriptor: [Attitude to Health] explode all trees 32744

#22 MeSH descriptor: [Qualitative Research] this term only 808

#23 MeSH descriptor: [Surveys and Questionnaires] this term only 22490

#24 MeSH descriptor: [Focus Groups] this term only 467

#25 MeSH descriptor: [Grounded Theory] this term only 7

#26 MeSH descriptor: [Interviews as Topic] this term only 1665

#27 (questionnaire* or survey* or interview* or focus group* or case stud* or observ*):ti,kw 95278

#28 (Qualitative or "grounded theory" or "phenomenological analysis" or "thematic analysis" or ethnograph* or "narrative analys*" or "phenomenological research") .ti,kw 19

#29 ((Clinician* or physician* or doctor* or GP* or practitioner* or nurse* or "health professional*" or patient*) near/5 (view* or experienc* or opinion* or attitude* or percep* or perceiv* or belie* or feel* or know* or understand*)):ti,kw 6930

#30 MeSH descriptor: [Patient Participation] this term only 1163

#31 MeSH descriptor: [Patient Acceptance of Health Care] this term only 2598

#32 MeSH descriptor: [Behavior] this term only 864

#33 MeSH descriptor: [Psychology, Social] explode all trees 20592

#34 willing*:ti,ab 3929

#35 {or #16-#34} 144067

#36 #15 and #35 97

**Cochrane Reviews (3)**
**Other Reviews (1)**
**Trials (93)**
Methods Studies (0)
Technology Assessments (0)
Economic Evaluations (0)
Cochrane Groups (0)

**Embase Classic+Embase 1947 to 2017 November 22**

1 exp *antibiotic agent/ or exp *antiinfective agent/ or exp *antiinflammatory agent/ or exp *antineoplastic agent/ or exp *antirheumatic agent/ or exp *cardiovascular agent/ or exp *central nervous system agents/ or exp *dermatological agent/ or exp *gastrointestinal agent/ or exp *hematologic agent/ or exp *agents affecting lipid metabolism/ or exp *radiosensitizing agent/ or exp *urinary tract agent/ or exp *agents acting on the genital system/ or exp *respiratory tract agent/ or exp *hypnotic agent/ or exp *central stimulant agent/ or exp *drug therapy/ (4996918)

2 *anaphylaxis/ (18117)

3 exp *adverse drug reaction/ (172131)

4 2 or 3 (188090)

5 1 and 4 (138653)

6 exp *Drug Hypersensitivity/ (23365)

7 exp *antibiotic agent/ae or exp *antiinfective agent/ae or exp *antiinflammatory agent/ae or exp *antineoplastic agents/ae or exp *antirheumatic agent/ae or exp *cardiovascular agent/ae or exp *central nervous system agents/ae or exp *dermatological agent/ae or exp *gastrointestinal agent/ae or exp *hematologic agent/ae or exp *central stimulant agent/ae or exp *drug therapy/ae (513723)

8 ((allerg* or sensitiv* or hypersensitiv* or intoleran* or anaphyla*) adj5 (drug* or medication* or medicine* or antibiotic* or penicillin*)).tw. (68847)

9 ((adrs or adverse) adj3 (drug* or medication* or medicine* or antibiotic* or penicillin*)).tw. (43921)

10 or/5-9 [Drug allergy] (726936)

11 ((Drug* or medication* or medicine*) adj3 (test* or tolerability or screen* or diagnos* or "re test*")).tw. (81775)

12 drug hypersensitivity/di [Diagnosis] (3492)

13 or/11-12 [Diagnosis or testing] (84811)

14 10 and 13 [Drug allergy and sensitivity testing] (12984)

15 exp Ethnic Group/ (122495)

16 attitude/ (59475)

17 health personnel attitude/ (73931)

18 exp attitude to health/ (98549)

19 Qualitative Research/ (49984)

20 Questionnaires/ (438829)

21 Focus Group/ (119844)

22 Grounded Theory/ (4588)

23 Interviews as topic/ (119795)

24 (questionnaire* or survey* or interview* or focus group* or case stud* or observ*).ti,kw. (471787)

25 (Qualitative or "grounded theory" or "phenomenological analysis" or "thematic analysis" or ethnograph* or "narrative analys*" or "phenomenological research").ti,kw. (54495)

26 ((Clinician* or physician* or doctor* or GP* or practitioner* or nurse* or "health professional*" or patient*) adj5 (view* or experienc* or opinion* or attitude* or percep* or perceiv* or belie* or feel* or know* or understand*)).tw. (547587)

27 patient participation/ (22942)

28 patient attitude/ (59808)

29 exp *Behavior/ (1370524)

30 social psychology/ (78081)

31 willing*.tw. (46582)

32 or/15-31 [Attitutudes or indicators of attitudes] (2926514)

33 14 and 32 [Drug allergy and attitudes or indicators of attitudes] (1108)

**Ovid MEDLINE(R) 1946 to November Week 2 2017**

1 exp anti-infective agents/ or exp anti-inflammatory agents/ or exp antineoplastic agents/ or exp antirheumatic agents/ or exp cardiovascular agents/ or exp central nervous system agents/ or exp dermatologic agents/ or exp gastrointestinal agents/ or exp hematologic agents/ or exp lipid regulating agents/ or exp pharmaceutical solutions/ or exp radiation-sensitizing agents/ or exp renal

agents/ or exp reproductive control agents/ or exp respiratory system agents/ or exp sleep aids, pharmaceutical/ or exp urological agents/ (5178346)

2 anaphylaxis/ (20491)

3 exp "Drug-Related Side Effects and Adverse Reactions"/ (110196)

4 2 or 3 (128050)

5 1 and 4 (67527)

6 exp *Drug Hypersensitivity/ (33262)

7 exp *anti-infective agents/ae or exp *anti-inflammatory agents/ae or exp *antineoplastic agents/ae or exp *antirheumatic agents/ae or exp *cardiovascular agents/ae or exp *central nervous system agents/ae or exp *dermatologic agents/ae or exp *gastrointestinal agents/ae or exp *hematologic agents/ae or exp *lipid regulating agents/ae or exp *pharmaceutical solutions/ae or exp *radiation-sensitizing agents/ae or exp *renal agents/ae or exp *reproductive control agents/ae or exp *respiratory system agents/ae or exp *sleep aids, pharmaceutical/ae or exp *stimulants, historical/ae or exp *urological agents/ae (262676)

8 ((allerg* or sensitiv* or hypersensitiv* or intoleran* or anaphyla*) adj3 (drug* or medication* or medicine*)).tw. (23343)

9 ((adrs or adverse) adj3 (reaction* or effect* or event*) adj3 (drug* or medication* or medicine*)).tw. (27162)

10 or/5-9 [Drug allergy] (349179)

11 ((Drug* or medication* or medicine*) adj3 (test* or tolerability or screen* or diagnos* or "re test*")).tw. (52480)

12 exp Drug Hypersensitivity/di (8590)

13 or/11-12 [Diagnosis or testing] (60406)

14 10 and 13 [Drug allergy and sensitivity testing] (12568)

15 exp Culture/ (155326)

16 exp Ethnic Groups/ (147157)

17 es.fs. (67043)

18 exp Ethics/ (144995)

19 attitude/ (46260)

20 "attitude of health personnel"/ (114611)

21 exp attitude to health/ (389148)

22 Qualitative Research/ (38929)

23 Questionnaires/ (418959)

24 Focus Group/ (25354)

25 Grounded Theory/ (764)

26 Interviews as topic/ (56996)

27 (questionnaire* or survey* or interview* or focus group* or case stud* or observ*).ti,kw. (339849)

28 (Qualitative or "grounded theory" or "phenomenological analysis" or "thematic analysis" or ethnograph* or "narrative analys*" or "phenomenological research").ti,kw. (33565)

29 ((Clinician* or physician* or doctor* or GP* or practitioner* or nurse* or "health professional*" or patient*) adj5 (view* or experienc* or opinion* or attitude* or percep* or perceiv* or belie* or feel* or know* or understand*)).tw. (345332)

30 Patient Participation/ (23570)

31 "Patient Acceptance of Health Care"/ (41387)

32 exp *Behavior/ (954235)

33 exp Psychology, Social/ (840271)

34 willing*.tw. (30239)

35 or/15-34 [Attitudes and indicators of attitude studies] (2867280)

36 14 and 35 [Drug allergy and sensitivity testing and Attitudes and indicators of attitude studies] (874)

**Ovid MEDLINE(R) Epub Ahead of Print November 15, 2017**

1 exp anti-infective agents/ or exp anti-inflammatory agents/ or exp antineoplastic agents/ or exp antirheumatic agents/ or exp cardiovascular agents/ or exp central nervous system agents/ or exp dermatologic agents/ or exp gastrointestinal agents/ or exp hematologic agents/ or exp lipid regulating agents/ or exp pharmaceutical solutions/ or exp radiation-sensitizing agents/ or exp renal agents/ or exp reproductive control agents/ or exp respiratory system agents/ or exp sleep aids, pharmaceutical/ or exp urological agents/ (0)

2 anaphylaxis/ (0)

3 exp "Drug-Related Side Effects and Adverse Reactions"/ (0)

4 2 or 3 (0)

5 1 and 4 (0)

6 exp *Drug Hypersensitivity/ (0)

7 exp *anti-infective agents/ae or exp *anti-inflammatory agents/ae or exp *antineoplastic agents/ae or exp *antirheumatic agents/ae or exp *cardiovascular agents/ae or exp *central nervous system agents/ae or exp *dermatologic agents/ae or exp *gastrointestinal agents/ae or exp *hematologic agents/ae or exp *lipid regulating agents/ae or exp *pharmaceutical solutions/ae or exp *radiation-sensitizing agents/ae or exp *renal agents/ae or exp *reproductive control agents/ae or exp *respiratory system agents/ae or exp *sleep aids, pharmaceutical/ae or exp *stimulants, historical/ae or exp *urological agents/ae (0)

8 ((allerg* or sensitiv* or hypersensitiv* or intoleran* or anaphyla*) adj3 (drug* or medication* or medicine*)).tw. (326)

9 ((adrs or adverse) adj3 (reaction* or effect* or event*) adj3 (drug* or medication* or medicine*)).tw. (559)

10 or/5-9 [Drug allergy] (873)

11 ((Drug* or medication* or medicine*) adj3 (test* or tolerability or screen* or diagnos* or "re test*")).tw. (1088)

12 exp Drug Hypersensitivity/di (0)

13 or/11-12 [Diagnosis or testing] (1088)

14 10 and 13 [Drug allergy and sensitivity testing] (63)

15 exp Culture/ (1)

16 exp Ethnic Groups/ (0)

17 es.fs. (0)

18 exp Ethics/ (0)

19 attitude/ (0)

20 "attitude of health personnel"/ (0)

21 exp attitude to health/ (0)

22 Qualitative Research/ (0)

23 "Questionnaires"/ (1)

24 Focus Group/ (0)

25 Grounded Theory/ (0)

26 Interviews as topic/ (0)

27 (questionnaire* or survey* or interview* or focus group* or case stud* or observ*).ti,kw. (9518)

28 (Qualitative or "grounded theory" or "phenomenological analysis" or "thematic analysis" or ethnograph* or "narrative analys*" or "phenomenological research").ti,kw. (1789)

29 ((Clinician* or physician* or doctor* or GP* or practitioner* or nurse* or "health professional*" or patient*) adj5 (view* or experienc* or opinion* or attitude* or percep* or perceiv* or belie* or feel* or know* or understand*)).tw. (8023)

30 Patient Participation/ (0)

31 "Patient Acceptance of Health Care"/ (0)

32 exp *Behavior/ (0)

33 exp Psychology, Social/ (1)

34 willing*.tw. (1021)

35 or/15-34 [Attitudes and indicators of attitude studies] (19159)

36 14 and 35 [Drug allergy and sensitivity testing and Attitudes and indicators of attitude studies] (3)

**Ovid MEDLINE(R) In-Process & Other Non-Indexed Citations November 22, 2017**

1 exp anti-infective agents/ or exp anti-inflammatory agents/ or exp antineoplastic agents/ or exp antirheumatic agents/ or exp cardiovascular agents/ or exp central nervous system agents/ or exp dermatologic agents/ or exp gastrointestinal agents/ or exp hematologic agents/ or exp lipid regulating agents/ or exp pharmaceutical solutions/ or exp radiation-sensitizing agents/ or exp renal agents/ or exp reproductive control agents/ or exp respiratory system agents/ or exp sleep aids, pharmaceutical/ or exp urological agents/ (225)

2 anaphylaxis/ (0)

3 exp "Drug-Related Side Effects and Adverse Reactions"/ (16)

4 2 or 3 (16)

5 1 and 4 (0)

6 exp *Drug Hypersensitivity/ (1)

7 exp *anti-infective agents/ae or exp *anti-inflammatory agents/ae or exp *antineoplastic agents/ae or exp *antirheumatic agents/ae or exp *cardiovascular agents/ae or exp *central nervous system agents/ae or exp *dermatologic agents/ae or exp *gastrointestinal agents/ae or exp *hematologic agents/ae or exp *lipid regulating agents/ae or exp *pharmaceutical solutions/ae or exp *radiation-sensitizing agents/ae or exp *renal agents/ae or exp *reproductive control agents/ae or exp *respiratory system agents/ae or exp *sleep aids, pharmaceutical/ae or exp *stimulants, historical/ae or exp *urological agents/ae (5)

8 ((allerg* or sensitiv* or hypersensitiv* or intoleran* or anaphyla*) adj3 (drug* or medication* or medicine*)).tw. (1933)

9 ((adrs or adverse) adj3 (reaction* or effect* or event*) adj3 (drug* or medication* or medicine*)).tw. (3554)

10 or/5-9 [Drug allergy] (5393)

11 ((Drug* or medication* or medicine*) adj3 (test* or tolerability or screen* or diagnos* or "re test*")).tw. (5873)

12 exp Drug Hypersensitivity/di (0)

13 or/11-12 [Diagnosis or testing] (5873)

14 10 and 13 [Drug allergy and sensitivity testing] (378)

15 exp Culture/ (5)

16 exp Ethnic Groups/ (7)

17 es.fs. (3)

18 exp Ethics/ (8)

19 attitude/ (0)

20 "attitude of health personnel"/ (1)

21 exp attitude to health/ (4)

22 Qualitative Research/ (0)

23 "Questionnaires"/ (8)

24 Focus Group/ (0)

25 Grounded Theory/ (0)

26 Interviews as topic/ (0)

27 (questionnaire* or survey* or interview* or focus group* or case stud* or observ*).ti,kw. (43275)

28 (Qualitative or "grounded theory" or "phenomenological analysis" or "thematic analysis" or ethnograph* or "narrative analys*" or "phenomenological research").ti,kw. (6471)

29 ((Clinician* or physician* or doctor* or GP* or practitioner* or nurse* or "health professional*" or patient*) adj5 (view* or experienc* or opinion* or attitude* or percep* or perceiv* or belie* or feel* or know* or understand*)).tw. (37937)

30 Patient Participation/ (1)

31 "Patient Acceptance of Health Care"/ (0)

32 exp *Behavior/ (69)

33 exp Psychology, Social/ (72)

34 willing*.tw. (4040)

35 or/15-34 [Attitudes and indicators of attitude studies] (86684)

36 14 and 35 [Drug allergy and sensitivity testing and Attitudes and indicators of attitude studies] (18)

**PsycINFO 1806 to November Week 2 2017**

1 exp ANTIBIOTICS/ or exp Anti Inflammatory Drugs/ or exp Antineoplastic Drugs/ or exp Drug Therapy/ or exp Drugs/ or exp Antidepressant Drugs/ or exp Prescription Drugs/ (336656)

2 exp "Side Effects (Drug)"/ or exp "Side Effects (Treatment)"/ or exp Anaphylactic Shock/ (54070)

3 1 and 2 (38792)

4 exp *Drug Sensitivity/ or exp DRUG ALLERGIES/ (2128)

5 ((allerg* or sensitiv* or hypersensitiv* or intoleran* or anaphyla*) adj5 (drug* or medication* or medicine* or antibiotic* or penicillin*)).tw. (2044)

6 ((adrs or adverse) adj3 (drug* or medication* or medicine* or antibiotic* or penicillin*)).tw. (2715)

7 or/3-6 [Drug allergy] (42329)

8 ((Drug* or medication* or medicine*) adj3 (test* or tolerability or screen* or diagnos* or "re test*")).tw. (9502)

9 exp DIAGNOSIS/ (160762)

10 exp SCREENING/ (22080)

11 or/8-10 [Testing] (185528)

12 7 and 11 [Drug allergy and sensitivity testing] (2387)

13 exp "CULTURE (ANTHROPOLOGICAL)"/ (90370)

14 exp "Racial and Ethnic Groups"/ (115331)

15 exp ETHICS/ (40351)

16 ATTITUDES/ (24447)

17 Health Attitudes/ (9550)

18 HEALTH PERSONNEL ATTITUDES/ (17491)

19 Qualitative Research/ (7642)

20 QUESTIONNAIRES/ (16772)

21 Group Discussion/ (3597)

22 Grounded Theory/ (3282)

23 Interviews/ (7982)

24 (questionnaire* or survey* or interview* or focus group* or case stud* or observ*).ti,id. (141481)

25 (Qualitative or "grounded theory" or "phenomenological analysis" or "thematic analysis" or ethnograph* or "narrative analys*" or "phenomenological research").ti,id. (41674)

26 ((Clinician* or physician* or doctor* or GP* or practitioner* or nurse* or "health professional*" or patient*) adj5 (view* or experienc* or opinion* or attitude* or percep* or perceiv* or belie* or feel* or know* or understand*)).ti,id. (23398)

27 Client Participation/ (1795)

28 *BEHAVIOR/ (15514)

29 exp Social Psychology/ (14168)

30 willing*.tw. (29722)

31 or/13-30 [Attitudes and indicators of attitude studies] (516584)

32 12 and 31 [drug allergy and attitudes and indicators of attitude studies] (114)

**PubMed (NLM) 1946 – present**

("drug allerg*" OR "drug hypersensitive*" and diagnos*) AND (pubstatusaheadofprint OR publisher[sb] OR pubmednotmedline[sb]) 118

# 14 #13 AND #5 1,481

Indexes=SCI-EXPANDED, SSCI, A&HCI, CPCI-S, CPCI-SSH, ESCI Timespan=1900-2017

# 13 #12 OR #11 OR #10 OR #9 OR #8 OR #7 OR #6 8,388,211

Indexes=SCI-EXPANDED, SSCI, A&HCI, CPCI-S, CPCI-SSH, ESCI Timespan=1900-2017

# 12 ts=willing* 62,960

Indexes=SCI-EXPANDED, SSCI, A&HCI, CPCI-S, CPCI-SSH, ESCI Timespan=1900-2017

# 11 ts=(Patient near (participation or accept*)) 56,370

Indexes=SCI-EXPANDED, SSCI, A&HCI, CPCI-S, CPCI-SSH, ESCI Timespan=1900-2017

# 10 ts=((Clinician* or physician* or doctor* or GP or GPs or practitioner* or nurse* or "health professional*" or patient*) near/5 (view* or experienc* or opinion* or attitude* or percep* or perceiv* or belie* or feel* or know* or understand*)) 325,704

Indexes=SCI-EXPANDED, SSCI, A&HCI, CPCI-S, CPCI-SSH, ESCI Timespan=1900-2017

# 9 ts=(Qualitative or "grounded theory" or "phenomenological analysis" or "thematic analysis" or ethnograph* or "narrative analys*" or "phenomenological research") 398,158

Indexes=SCI-EXPANDED, SSCI, A&HCI, CPCI-S, CPCI-SSH, ESCI Timespan=1900-2017

# 8 ts=(questionnaire* or survey* or interview* or focus group* or case stud* or observ*)

7,766,406 Indexes=SCI-EXPANDED, SSCI, A&HCI, CPCI-S, CPCI-SSH, ESCI Timespan=1900-2017

# 7 ts=Ethic* 180,338

Indexes=SCI-EXPANDED, SSCI, A&HCI, CPCI-S, **CPCI**-SSH, ESCI Timespan=1900-2017

# 6 ts=(Ethnic Group*) 55,302

# 5 #4 AND #3 4,559

# 4 ts=((Drug* or medication* or medicine*) near/3 (test* or tolerability or screen* or diagnos* or "re test*")) 62,586 Indexes=SCI-EXPANDED, SSCI, A&HCI, CPCI-S, CPCI-SSH, ESCI Timespan=1900-2017

# 3 #2 OR #1 72,848 Indexes=SCI-EXPANDED, SSCI, A&HCI, CPCI-S, CPCI-SSH, ESCI Timespan=1900-2017

# 2 ts=((adrs or adverse) near/3 (drug* or medication* or medicine* or antibiotic* or penicillin*)) 33,234

Indexes=SCI-EXPANDED, SSCI, A&HCI, CPCI-S, CPCI-SSH, ESCI Timespan=1900-2017

# 1 ts=((allerg* or sensitiv* or hypersensitiv* or intoleran* or anaphyla*) near/5 (drug* or medication* or medicine* or antibiotic* or penicillin*)) 41,053

Indexes=SCI-EXPANDED, SSCI, A&HCI, CPCI-S, CPCI-SSH, ESCI Timespan=1900-2017

**Search 3: Influences on clinicians' prescribing behaviour and patients use/consumption of drugs in relation to drug allergy**

**Applied Social Sciences Index and Abstracts (ASSIA) (ProQuest) 1987- present**

((MAINSUBJECT.EXACT.EXPLODE("Prescriptions") AND (MAINSUBJECT.EXACT.EXPLODE("Attitudes") OR MAINSUBJECT.EXACT("Patient satisfaction") OR MAINSUBJECT.EXACT("Health education"))) OR ti,ab((prescribe OR prescription) NEAR/8 ((Clinician OR physician OR doctor OR GP OR practitioner OR nurse OR "health professional*" OR patient) NEAR/6 (view OR experience OR opinion OR attitude OR perception OR perceive OR belief OR believe OR feel OR know OR understand OR fear OR expectation OR anxiety OR anxious OR influence OR behaviour OR behavior OR barrier OR risk OR decision OR willing OR unwilling))) OR ti,ab((Manage OR understand OR view OR experience OR opinion OR attitude OR perception OR perceive OR believe OR belief OR feel OR know OR understand OR fear OR expectation OR anxiety OR anxious) NEAR/6 ("drug hypersensitive" OR "drug hypersensitivity" OR "drug allerg*")) OR ti,ab("Treated with" NEAR/5 (medication OR drug OR antibiotic))) AND (MAINSUBJECT.EXACT.EXPLODE("Qualitative research") OR ti,ab(Qualitative OR "grounded theory" OR "phenomenological analysis" OR "thematic analysis" OR ethnograph* OR "narrative analys*" OR "phenomenological research") OR ti,ab(interview* OR questionnaire* OR "focus group*") OR MAINSUBJECT.EXACT.EXPLODE("Questionnaires")) 133

**CINAHL (EBSCO) 1981- present**

| | | |
|---|---|---|
| S25 | S20 AND S24 | 112 |
| S24 | S21 OR S22 OR S23 | 375,670 |
| S23 | (MH "Questionnaires+") | 225,854 |
| S22 | TI ( (interview* or questionnaire* or "focus group*") ) OR AB ( (interview* or questionnaire* or "focus group*") ) | 210,815 |
| S21 | TI ( (Qualitative or "grounded theory" or "phenomenological analysis" or "thematic analysis" or ethnograph* or "narrative analys*" or "phenomenological research") ) OR AB ( (Qualitative or "grounded theory" or "phenomenological analysis" or "thematic analysis" or ethnograph* or "narrative analys*" or "phenomenological research") ) | 74,299 |
| S20 | S10 AND S19 | 566 |
| S19 | S16 OR S17 OR S18 | 3,797 |
| S18 | TI ( ((Manage* or understand* or view* or experienc* or opinion* or attitude* or percep* or perceiv* or belie* or feel* or know* or understand* or fear* or expectation* or anxiety or anxious) N6 (drug hypersensitiv* or drug allerg*)) ) OR AB ( ((Manage* or understand* or view* or experienc* or opinion* or attitude* or percep* or perceiv* or belie* or feel* or know* or understand* or fear* or expectation* or anxiety or anxious) N6 (drug hypersensitiv* or drug allerg*)) ) | 64 |
| S17 | TI ( ((prescrib* or prescrip*) N8 ((Clinician* or physician* or doctor* or GP* or practitioner* or nurse* or "health professional*" or patient*) N6 (view* or experienc* or opinion* or attitude* or percep* or perceiv* or belie* or feel* or know* or understand* or fear* or expectation* or anxiety or anxious or influenc* or behavi* or barrier* or risk* or decision* or willing* or unwilling*))) ) OR AB ( ((prescrib* or prescrip*) N8 ((Clinician* | 2,467 |

| | | |
|---|---|---|
| | or physician* or doctor* or GP* or practitioner* or nurse* or "health professional*" or patient*) N6 (view* or experienc* or opinion* or attitude* or percep* or perceiv* or belie* or feel* or know* or understand* or fear* or expectation* or anxiety or anxious or influenc* or behavi* or barrier* or risk* or decision* or willing* or unwilling*))) ) | |
| S16 | S14 AND S15 | 1,427 |
| S15 | (MM "Practice Patterns" OR MM "Patient Satisfaction" OR MM "Attitude of Health Personnel+" OR MM "Attitude to Health" OR MM "Health Knowledge") | 64,070 |
| S14 | S12 OR S13 | 110,597 |
| S13 | (MH "Prescriptions, Drug") OR (MH "Drugs, Prescription") OR (MH Prescribing patterns) OR (MH Inappropriate prescribing) | 19,783 |
| S12 | S1 AND S11 | 93,039 |
| S11 | TX Administration and dosage* | 158,993 |
| S10 | S5 OR S6 OR S7 OR S8 OR S9 | 73,953 |
| S9 | TI ( ((adrs or adverse) N3 (drug* or medication* or medicine* or antibiotic* or penicillin*)) ) OR AB ( ((adrs or adverse) N3 (drug* or medication* or medicine* or antibiotic* or penicillin*)) ) | 5,558 |
| S8 | TI ( ((allerg* or sensitiv* or hypersensitiv* or intoleran* or anaphyla*) N5 (drug* or medication* or medicine* or antibiotic* or penicillin*)) ) OR AB ( ((allerg* or sensitiv* or hypersensitiv* or intoleran* or anaphyla*) N5 (drug* or medication* or medicine* or antibiotic* or penicillin*)) ) | 2,763 |
| S7 | (MH "Antiinfective Agents/AE" or MH "Antiinfective Agents/AE" or MH "Antiinflammatory Agents+/AE" or MH "Antineoplastic Agents+/AE" or MH "Antirheumatic Agents+/AE" or MH "Cardiovascular Agents+/AE" or MH "Central Nervous System Agents+/AE" or MH "Dermatologic Agents+/AE" or MH "Gastrointestinal Agents+ AE" or MH "Hematologic Agents+/AE" or MH " Antilipemic Agents+ AE" or MH "Radiation-Sensitizing Agents"+/AE" or MH "Renal Agents + AE" or MH "Reproductive Control Agent+/AE" OR MH "Drug Therapy/AE) | 59,113 |
| S6 | (MM "Drug Hypersensitivity+") | 2,179 |
| S5 | S1 AND S4 | 8,658 |
| S4 | S2 OR S3 | 11,154 |
| S3 | (MM "Adverse Drug Event+") | 8,934 |
| S2 | (MH "Anaphylaxis") | 2,253 |
| S1 | (MH "Antibiotics+" or MH "Antiinflammatory Agents+" or MH "Antineoplastic Agents" or MH "Antirheumatic Agents" or MH "Cardiovascular Agents+" or MH "Central Nervous System Agents+" or MH "Dermatologic Agents+" or MH "Gastrointestinal Agents+" or MH "Hematologic Agents+" or MH "Antilipemic Agents+" or MH "Radiation- | 342,534 |

Sensitizing Agents+" or MH "Renal Agents" or MH "Reproductive Control Agents+" OR MH "Drug Therapy+")

**Cochrane library**

- **Cochrane Database of Systematic Reviews : Issue 11 of 12, November 2017**
- **Cochrane Central Register of Controlled Trials : Issue 10 of 12, October 2017**
- **Database of Abstracts of Reviews of Effect : Issue 2 of 4, April 2015**
- **NHS Economic Evaluation Database : Issue 2 of 4, April 2015**

| ID | Search | Hits |
|---|---|---|
| #1 | MeSH descriptor: [Anti-Infective Agents] explode all trees | 27900 |
| #2 | MeSH descriptor: [Anti-Inflammatory Agents] explode all trees | 12708 |
| #3 | MeSH descriptor: [Antineoplastic Agents] explode all trees | 12490 |
| #4 | MeSH descriptor: [Antirheumatic Agents] explode all trees | 9607 |
| #5 | MeSH descriptor: [Cardiovascular Agents] explode all trees | 21520 |
| #6 | MeSH descriptor: [Central Nervous System Agents] explode all trees | 48207 |
| #7 | MeSH descriptor: [Dermatologic Agents] explode all trees | 2887 |
| #8 | MeSH descriptor: [Gastrointestinal Agents] explode all trees | 7857 |
| #9 | MeSH descriptor: [Hematologic Agents] explode all trees | 12513 |
| #10 | MeSH descriptor: [Lipid Regulating Agents] explode all trees | 6583 |
| #11 | MeSH descriptor: [Pharmaceutical Solutions] explode all trees | 4117 |
| #12 | MeSH descriptor: [Radiation-Sensitizing Agents] explode all trees | 863 |
| #13 | MeSH descriptor: [Renal Agents] explode all trees | 403 |
| #14 | MeSH descriptor: [Reproductive Control Agents] explode all trees | 4709 |
| #15 | MeSH descriptor: [Respiratory System Agents] explode all trees | 6628 |
| #16 | MeSH descriptor: [Sleep Aids, Pharmaceutical] explode all trees | 26 |
| #17 | MeSH descriptor: [Urological Agents] explode all trees | 104 |
| #18 | {or #1-#17} | 154811 |
| #19 | MeSH descriptor: [Anaphylaxis] explode all trees | 183 |

#20 MeSH descriptor: [Drug-Related Side Effects and Adverse Reactions] explode all trees 3060

#21 #19 or #20 3218

#22 #18 and #21 1473

#23 MeSH descriptor: [Drug Hypersensitivity] this term only 446

#24 ((allerg* or sensitiv* or hypersensitiv* or intoleran* or anaphyla*) near/5 (drug* or medication* or medicine* or antibiotic* or penicillin*)):ti,ab 3321

#25 ((adrs or adverse) near/3 (drug* or medication* or medicine* or antibiotic* or penicillin*)):ti,ab 5223

#26 #22 or #23 or 24 or #25 185594

#27 MeSH descriptor: [Drug Prescriptions] explode all trees 861

#28 MeSH descriptor: [Practice Patterns, Physicians'] this term only 1317

#29 MeSH descriptor: [Patient Satisfaction] this term only 10824

#30 MeSH descriptor: [Patient Preference] this term only 631

#31 MeSH descriptor: [Attitude of Health Personnel] this term only 1845

#32 MeSH descriptor: [Attitude to Health] this term only 2963

#33 MeSH descriptor: [Health Knowledge, Attitudes, Practice] this term only 5247

#34 {or #27-#33} 21659

#35 #26 and #34 3265

#36 ((prescrib* or prescrip*) near/8 ((Clinician* or physician* or doctor* or GP* or practitioner* or nurse* or "health professional*" or patient*) near/6 (view* or experienc* or opinion* or attitude* or percep* or perceiv* or belie* or feel* or know* or understand* or fear* or expectation* or anxiety or anxious or influenc* or behavi* or barrier* or risk* or decision* or willing* or unwilling*))):ti,ab 826

#37 ((Manage* or understand* or view* or experienc* or opinion* or attitude* or percep* or perceiv* or belie* or feel* or know* or understand* or fear* or expectation* or anxiety or anxious) near/6 (drug hypersensitiv* or drug allerg*)):ti,ab 12

#38 ("Treated with" near/5 (medication* or drug* or antibiotic*)):ti,ab 3522

#39 {or #35-#38} 7567

#40 #26 and #39 4282

#41 MeSH descriptor: [Qualitative Research] explode all trees 818

#42 (Qualitative or "grounded theory" or "phenomenological analysis" or "thematic analysis" or ethnograph* or "narrative analys*" or "phenomenological research"):ti,ab 7757

#43 (interview* or questionnaire* or "focus group*"):ti,ab 66124

#44 MeSH descriptor: [Surveys and Questionnaires] this term only 22566

#45 {or #41-#44} 80773

#46 #40 and #45 1113

**Embase Classic+Embase 1947 to 2017 November 01**

1 exp antibiotic agent/ or exp antiinfective agent/ or exp *antiinflammatory agent/ or exp *antineoplastic agent/ or exp *antirheumatic agent/ or exp *cardiovascular agent/ or exp *central nervous system agents/ or exp *dermatological agent/ or exp *gastrointestinal agent/ or exp *hematologic agent/ or exp *agents affecting lipid metabolism/ or exp *radiosensitizing agent/ or exp *urinary tract agent/ or exp *agents acting on the genital system/ or exp *respiratory tract agent/ or exp *hypnotic agent/ or exp *central stimulant agent/ or exp *drug therapy/ (6397954)

2 *anaphylaxis/ (18080)

3 exp adverse drug reaction/ (474879)

4 2 or 3 (489710)

5 1 and 4 (340366)

6 exp *drug hypersensitivity/ (23314)

7 exp *antibiotic agent/ae or exp *antiinfective agent/ae or exp *antiinflammatory agent/ae or exp *antineoplastic agents/ae or exp *antirheumatic agent/ae or exp *cardiovascular agent/ae or exp *central nervous system agents/ae or exp *dermatological agent/ae or exp *gastrointestinal agent/ae or exp *hematologic agent/ae or exp *central stimulant agent/ae or exp *drug therapy/ae (512310)

8 ((allerg* or sensitiv* or hypersensitiv* or intoleran* or anaphyla*) adj5 (drug* or medication* or medicine* or antibiotic* or penicillin*)).tw. (68565)

9 ((adrs or adverse) adj3 (drug* or medication* or medicine* or antibiotic* or penicillin*)).tw. (43639)

10 or/5-9 [Drug allergy] (824462)

11 ad.fs. (453617)

12 1 and 11 (241787)

13 exp *prescription/ (33337)

14 12 or 13 (274715)

15 *physician attitude/ or *patient satisfaction/ or *patient preference/ (41760)

16 exp *health personnel attitude/ or *attitude to health/ or *attitude/ (144011)

17 15 or 16 [Clinical or patient attitudes EMTREE] (166280)

18 14 and 17 [Prescribing drugs AND Clinician patient attitudes EMTREE] (1627)

19 ((prescrib* or prescrip*) adj8 ((Clinician* or physician* or doctor* or GP* or practitioner* or nurse* or "health professional*" or patient*) adj6 (view* or experienc* or opinion* or attitude* or percep* or perceiv* or belie* or feel* or know* or understand* or fear* or expectation* or anxiety or anxious or influenc* or behavi* or barrier* or risk* or decision* or willing* or unwilling*))).tw. (11767)

20 ((Manage* or understand* or view* or experienc* or opinion* or attitude* or percep* or perceiv* or belie* or feel* or know* or understand* or fear* or expectation* or anxiety or anxious) adj6 (drug hypersensitiv* or drug allerg*)).tw. (413)

21 ("Treated with" adj5 (medication* or drug* or antibiotic*)).tw. (45945)

22 or/18-21 [Prescribing drugs AND Clinician patient attitudes EMTREE or textwords] (59281)

23 10 and 22 (8229)

24 qualitative research/ (49499)

25 (Qualitative or "grounded theory" or "phenomenological analysis" or "thematic analysis" or ethnograph* or "narrative analys*" or "phenomenological research").tw. (240168)

26 (interview* or questionnaire* or "focus group*").tw. (910486)

27 exp questionnaire/ (570836)

28 or/24-27 [Qualitative studies] (1200410)

29 23 and 28 (603)

**Ovid MEDLINE(R) 1946 to November Week 1 2017**

1 exp anti-infective agents/ or exp anti-inflammatory agents/ or exp antineoplastic agents/ or exp antirheumatic agents/ or exp cardiovascular agents/ or exp central nervous system agents/ or exp dermatologic agents/ or exp gastrointestinal agents/ or exp hematologic agents/ or exp lipid regulating agents/ or exp pharmaceutical solutions/ or exp radiation-sensitizing agents/ or exp renal agents/ or exp reproductive control agents/ or exp respiratory system agents/ or exp sleep aids, pharmaceutical/ or exp urological agents/ (5175320)

2 anaphylaxis/ (20475)

3 exp "Drug-Related Side Effects and Adverse Reactions"/ (110139)

4 2 or 3 (127980)

5 1 and 4 (67497)

6 exp *Drug Hypersensitivity/ (33244)

7 exp *anti-infective agents/ae or exp *anti-inflammatory agents/ae or exp *antineoplastic agents/ae or exp *antirheumatic agents/ae or exp *cardiovascular agents/ae or exp *central nervous system agents/ae or exp *dermatologic agents/ae or exp *gastrointestinal agents/ae or exp *hematologic agents/ae or exp *lipid regulating agents/ae or exp *pharmaceutical solutions/ae or exp *radiation-sensitizing agents/ae or exp *renal agents/ae or exp *reproductive control agents/ae or exp *respiratory system agents/ae or exp *sleep aids, pharmaceutical/ae or exp *stimulants, historical/ae or exp *urological agents/ae (262544)

8 ((allerg* or sensitiv* or hypersensitiv* or intoleran* or anaphyla*) adj5 (drug* or medication* or medicine* or antibiotic* or penicillin*)).tw. (43829)

9 ((adrs or adverse) adj3 (drug* or medication* or medicine* or antibiotic* or penicillin*)).tw. (25392)

10 or/5-9 [Drug allergy] (365254)

11 ad.fs. (1381689)

12 1 and 11 (900710)

13 exp Drug Prescriptions/ (31221)

14 12 or 13 [Drug prescription MESH] (927825)

15 *Physician's Practice Patterns/ or *patient satisfaction/ or *patient preference/ (64793)

16 exp *"Attitude of Health Personnel"/ or *attitude to health/ or *health knowledge, attitudes, practice/ (168008)

17 15 or 16 [Clinical or patient attitudes MESH] (227485)

18 14 and 17 [Prescribing drugs AND Clinician OR patient attitudes MESH] (8590)

19 ((prescrib* or prescrip*) adj8 ((Clinician* or physician* or doctor* or GP* or practitioner* or nurse* or "health professional*" or patient*) adj6 (view* or experienc* or opinion* or attitude* or percep* or perceiv* or belie* or feel* or know* or understand* or fear* or expectation* or anxiety or anxious or influenc* or behavi* or barrier* or risk* or decision* or willing* or unwilling*))).tw. (6841)

20 ((Manage* or understand* or view* or experienc* or opinion* or attitude* or percep* or perceiv* or belie* or feel* or know* or understand* or fear* or expectation* or anxiety or anxious) adj6 (drug hypersensitiv* or drug allerg*)).tw. (198)

21 ("Treated with" adj5 (medication* or drug* or antibiotic*)).tw. (27961)

22 or/18-21 [Prescribing drugs AND Clinician patient attitudes MESH or textwords] (42663)

23 10 and 22 (4363)

24 Qualitative Research/ (38842)

25 (Qualitative or "grounded theory" or "phenomenological analysis" or "thematic analysis" or ethnograph* or "narrative analys*" or "phenomenological research").tw. (171661)

26 (interview* or questionnaire* or "focus group*").tw. (633340)

27 exp "Surveys and Questionnaires"/ (932159)

28 or/24-27 [Qualitative studies] (1381573)

29 23 and 28 (522)

**Ovid MEDLINE(R) Epub Ahead of Print November 09, 2017**

1 exp anti-infective agents/ or exp anti-inflammatory agents/ or exp antineoplastic agents/ or exp antirheumatic agents/ or exp cardiovascular agents/ or exp central nervous system agents/ or exp dermatologic agents/ or exp gastrointestinal agents/ or exp hematologic agents/ or exp lipid regulating agents/ or exp pharmaceutical solutions/ or exp radiation-sensitizing agents/ or exp renal agents/ or exp reproductive control agents/ or exp respiratory system agents/ or exp sleep aids, pharmaceutical/ or exp urological agents/ (0)

2 anaphylaxis/ (0)

3 exp "Drug-Related Side Effects and Adverse Reactions"/ (0)

4 2 or 3 (0)

5 1 and 4 (0)

6 exp *Drug Hypersensitivity/ (0)

7 exp *anti-infective agents/ae or exp *anti-inflammatory agents/ae or exp *antineoplastic agents/ae or exp *antirheumatic agents/ae or exp *cardiovascular agents/ae or exp *central nervous system agents/ae or exp *dermatologic agents/ae or exp *gastrointestinal agents/ae or exp *hematologic agents/ae or exp *lipid regulating agents/ae or exp *pharmaceutical solutions/ae or exp *radiation-sensitizing agents/ae or exp *renal agents/ae or exp *reproductive control agents/ae or exp *respiratory system agents/ae or exp *sleep aids, pharmaceutical/ae or exp *stimulants, historical/ae or exp *urological agents/ae (0)

8 ((allerg* or sensitiv* or hypersensitiv* or intoleran* or anaphyla*) adj5 (drug* or medication* or medicine* or antibiotic* or penicillin*)).tw. (546)

9 ((adrs or adverse) adj3 (drug* or medication* or medicine* or antibiotic* or penicillin*)).tw. (540)

10 or/5-9 [Drug allergy] (1068)

11 ad.fs. (0)

12 1 and 11 (0)

13 exp Drug Prescriptions/ (0)

14 12 or 13 [Drug prescription MESH] (0)

15 *Physician's Practice Patterns/ or *patient satisfaction/ or *patient preference/ (0)

16 exp *"Attitude of Health Personnel"/ or *attitude to health/ or *health knowledge, attitudes, practice/ (0)

17 15 or 16 [Clinical or patient attitudes MESH] (0)

18 14 and 17 [Prescribing drugs AND Clinician OR patient attitudes MESH] (0)

19 ((prescrib* or prescrip*) adj8 ((Clinician* or physician* or doctor* or GP* or practitioner* or nurse* or "health professional*" or patient*) adj6 (view* or experienc* or opinion* or attitude* or percep* or perceiv* or belie* or feel* or know* or understand* or fear* or expectation* or anxiety or anxious or influenc* or behavi* or barrier* or risk* or decision* or willing* or unwilling*))).tw. (179)

20 ((Manage* or understand* or view* or experienc* or opinion* or attitude* or percep* or perceiv* or belie* or feel* or know* or understand* or fear* or expectation* or anxiety or anxious) adj6 (drug hypersensitiv* or drug allerg*)).tw. (4)

21 ("Treated with" adj5 (medication* or drug* or antibiotic*)).tw. (393)

22 or/18-21 [Prescribing drugs AND Clinician patient attitudes MESH or textwords] (575)

23 10 and 22 (18)

24 Qualitative Research/ (0)

25 (Qualitative or "grounded theory" or "phenomenological analysis" or "thematic analysis" or ethnograph* or "narrative analys*" or "phenomenological research").tw. (7130)

26 (interview* or questionnaire* or "focus group*").tw. (17666)

27 exp "Surveys and Questionnaires"/ (2)

28 or/24-27 [Qualitative studies] (21216)

29 23 and 28 (2)

**Ovid MEDLINE(R) In-Process & Other Non-Indexed Citations November 09, 2017**

1 exp anti-infective agents/ or exp anti-inflammatory agents/ or exp antineoplastic agents/ or exp antirheumatic agents/ or exp cardiovascular agents/ or exp central nervous system agents/ or exp dermatologic agents/ or exp gastrointestinal agents/ or exp hematologic agents/ or exp lipid regulating agents/ or exp pharmaceutical solutions/ or exp radiation-sensitizing agents/ or exp renal

agents/ or exp reproductive control agents/ or exp respiratory system agents/ or exp sleep aids, pharmaceutical/ or exp urological agents/ (225)

2 anaphylaxis/ (0)

3 exp "Drug-Related Side Effects and Adverse Reactions"/ (16)

4 2 or 3 (16)

5 1 and 4 (0)

6 exp *Drug Hypersensitivity/ (1)

7 exp *anti-infective agents/ae or exp *anti-inflammatory agents/ae or exp *antineoplastic agents/ae or exp *antirheumatic agents/ae or exp *cardiovascular agents/ae or exp *central nervous system agents/ae or exp *dermatologic agents/ae or exp *gastrointestinal agents/ae or exp *hematologic agents/ae or exp *lipid regulating agents/ae or exp *pharmaceutical solutions/ae or exp *radiation-sensitizing agents/ae or exp *renal agents/ae or exp *reproductive control agents/ae or exp *respiratory system agents/ae or exp *sleep aids, pharmaceutical/ae or exp *stimulants, historical/ae or exp *urological agents/ae (5)

8 ((allerg* or sensitiv* or hypersensitiv* or intoleran* or anaphyla*) adj5 (drug* or medication* or medicine* or antibiotic* or penicillin*)).tw. (3425)

9 ((adrs or adverse) adj3 (drug* or medication* or medicine* or antibiotic* or penicillin*)).tw. (3256)

10 or/5-9 [Drug allergy] (6562)

11 ad.fs. (13)

12 1 and 11 (7)

13 exp Drug Prescriptions/ (1)

14 12 or 13 [Drug prescription MESH] (8)

15 *Physician's Practice Patterns/ or *patient satisfaction/ or *patient preference/ (1)

16 exp *"Attitude of Health Personnel"/ or *attitude to health/ or *health knowledge, attitudes, practice/ (1)

17 15 or 16 [Clinical or patient attitudes MESH] (2)

18 14 and 17 [Prescribing drugs AND Clinician OR patient attitudes MESH] (0)

19 ((prescrib* or prescrip*) adj8 ((Clinician* or physician* or doctor* or GP* or practitioner* or nurse* or "health professional*" or patient*) adj6 (view* or experienc* or opinion* or attitude* or percep* or perceiv* or belie* or feel* or know* or understand* or fear* or expectation* or anxiety or anxious or influenc* or behavi* or barrier* or risk* or decision* or willing* or unwilling*))).tw. (864)

20 ((Manage* or understand* or view* or experienc* or opinion* or attitude* or percep* or perceiv* or belie* or feel* or know* or understand* or fear* or expectation* or anxiety or anxious) adj6 (drug hypersensitiv* or drug allerg*)).tw. (31)

21 ("Treated with" adj5 (medication* or drug* or antibiotic*)).tw. (2487)

22 or/18-21 [Prescribing drugs AND Clinician patient attitudes MESH or textwords] (3374)

23 10 and 22 (157)

24 Qualitative Research/ (0)

25 (Qualitative or "grounded theory" or "phenomenological analysis" or "thematic analysis" or ethnograph* or "narrative analys*" or "phenomenological research").tw. (27550)

26 (interview* or questionnaire* or "focus group*").tw. (74100)

27 exp "Surveys and Questionnaires"/ (20)

28 or/24-27 [Qualitative studies] (90355)

29 23 and 28 (18)

**PsycINFO 1806 to October Week 4 2017**

1 exp ANTIBIOTICS/ or exp Anti Inflammatory Drugs/ or exp Antineoplastic Drugs/ or exp Drug Therapy/ or exp Drugs/ or exp Antidepressant Drugs/ or exp Prescription Drugs/ (336070)

2 exp "Side Effects (Drug)"/ or exp "Side Effects (Treatment)"/ or exp Anaphylactic Shock/ or exp DRUG ALLERGIES/ (54027)

3 1 and 2 (38765)

4 exp *Drug Sensitivity/ (2080)

5 ((allerg* or sensitiv* or hypersensitiv* or intoleran* or anaphyla*) adj5 (drug* or medication* or medicine* or antibiotic* or penicillin*)).tw. (2043)

6 ((adrs or adverse) adj3 (drug* or medication* or medicine* or antibiotic* or penicillin*)).tw. (2704)

7 or/3-6 [Drug allergy] (42285)

8 exp "Prescribing (Drugs)"/ (3258)

9 exp DRUG DOSAGES/ (10720)

10 8 or 9 [Drug prescription indexing] (13826)

11 client satisfaction/ (4967)

12 Client Attitudes/ (15447)

13 Preferences/ (16173)

14 exp *Health Personnel Attitudes/ (17932)

15 exp *ATTITUDES/ (264680)

16 exp *Health Knowledge/ (5555)

17 or/11-16 [Clinical or patient attitudes Indexing] (285582)

18 10 and 17 (426)

19 ((prescrib* or prescrip*) adj8 ((Clinician* or physician* or doctor* or GP* or practitioner* or nurse* or "health professional*" or patient*) adj6 (view* or experienc* or opinion* or attitude* or percep* or perceiv* or belie* or feel* or know* or understand* or fear* or expectation* or anxiety or anxious or influenc* or behavi* or barrier* or risk* or decision* or willing* or unwilling*))).tw. (1951)

20 ((Manage* or understand* or view* or experienc* or opinion* or attitude* or percep* or perceiv* or belie* or feel* or know* or understand* or fear* or expectation* or anxiety or anxious) adj6 (drug hypersensitiv* or drug allerg*)).tw. (10)

21 ("Treated with" adj5 (medication* or drug* or antibiotic*)).tw. (2694)

22 or/18-20 [Prescribing drugs AND Clinician patient attitudes Indexing or textwords] (2258)

23 7 and 22 (236)

24 exp Qualitative Research/ (7632)

25 (Qualitative or "grounded theory" or "phenomenological analysis" or "thematic analysis" or ethnograph* or "narrative analys*" or "phenomenological research").tw. (168926)

26 (interview* or questionnaire* or "focus group*").tw. (503466)

27 exp QUESTIONNAIRES/ (17023)

28 or/24-27 [Qualitative studies] (586393)

29 23 and 28 (35)

**Web of Science - Clarivate Analytics**

- **Arts & Humanities Citation Index (Clarivate Analytics Web of Science) 1975-present**
- **Conference Proceedings Citation Index- Science (Clarivate Analytics Web of Science) 1990-present**
- **Conference Proceedings Citation Index- Social Science & Humanities (Clarivate Analytics Web of Science) 1990-present**
- **Sciences Citation Index (Clarivate Analytics Web of Science) 1900-present**
- **Social Sciences Citation Index (Clarivate Analytics Web of Science) 1900-present**
- **Web of Science Core Collection: Citation Indexes (Clarivate Analytics) 1900-present**
- **Emerging Sources Citation Index (ESCI) --2015-present**

# 9   #8 AND #7 AND #3  161

Indexes=SCI-EXPANDED, SSCI, A&HCI, CPCI-S, CPCI-SSH, ESCI Timespan=1900-2017

# 8   ts=(Qualitative or "grounded theory" or "phenomenological analysis" or "thematic analysis" or ethnograph* or "narrative analys*" or "phenomenological research" or interview* or questionnaire* or "focus group*") 1,134,936

Indexes=SCI-EXPANDED, SSCI, A&HCI, CPCI-S, CPCI-SSH, ESCI Timespan=1900-2017

# 7   #6 OR #5 OR #4   29,357

Indexes=SCI-EXPANDED, SSCI, A&HCI, CPCI-S, CPCI-SSH, ESCI Timespan=1900-2017

# 6   TS=("Treated with" NEAR/5 (medication* or drug* or antibiotic*))  22,163

Indexes=SCI-EXPANDED, SSCI, A&HCI, CPCI-S, CPCI-SSH, ESCI Timespan=1900-2017

# 5 TS=((Manage* or understand* or view* or experienc* or opinion* or attitude* or percep* or perceiv* or belie* or feel* or know* or understand* or fear* or expectation* or anxiety or anxious) near/6 ("drug hypersensitiv*" or "drug allerg*")) 200

Indexes=SCI-EXPANDED, SSCI, A&HCI, CPCI-S, CPCI-SSH, ESCI Timespan=1900-2017

# 4   ts=((prescrib* or prescrip*) near/8 ((Clinician* or physician* or doctor* or GP or GPs or practitioner* or nurse* or "health professional*" or patient*) near/6 (view* or experienc* or opinion* or attitude* or percep* or perceiv* or belie* or feel* or know* or understand* or fear* or expectation* or anxiety or anxious or influenc* or behavi* or barrier* or risk* or decision* or willing* or unwilling*))) 7,063

Indexes=SCI-EXPANDED, SSCI, A&HCI, CPCI-S, CPCI-SSH, ESCI Timespan=1900-2017

# 3   #2 OR #1   72,862

Indexes=SCI-EXPANDED, SSCI, A&HCI, CPCI-S, CPCI-SSH, ESCI Timespan=1900-2017

# 2 ts=((adrs or adverse) near/3 (drug* or medication* or medicine* or antibiotic* or penicillin*))

Indexes=SCI-EXPANDED, SSCI, A&HCI, CPCI-S, CPCI-SSH, ESCI Timespan=1900-2017

33,243

# 1   ts=((allerg* or sensitiv* or hypersensitiv* or intoleran* or anaphyla*) near/5 (drug* or medication* or medicine* or antibiotic* or penicillin*))  41,058

Indexes=SCI-EXPANDED, SSCI, A&HCI, CPCI-S, CPCI-SSH, ESCI Timespan=1900-2017
